# Impact of aromatase inhibitor treatment on global gene expression and its association with antiproliferative response in ER+ breast cancer in postmenopausal patients

**DOI:** 10.1186/s13058-019-1223-z

**Published:** 2019-12-31

**Authors:** Qiong Gao, Elena López-Knowles, Maggie Chon U. Cheang, James Morden, Ricardo Ribas, Kally Sidhu, David Evans, Vera Martins, Andrew Dodson, Anthony Skene, Chris Holcombe, Elizabeth Mallon, Abigail Evans, Judith M. Bliss, John Robertson, Ian Smith, Lesley-Ann Martin, Mitch Dowsett, on behalf of the POETIC Trial Management Group and Trialists

**Affiliations:** 1 Breast Cancer Now Research Centre, ICR, London, UK; 2 Ralph Lauren Centre for Breast Cancer Research, Royal Marsden Hospital, London, UK; 3 Clinical Trials and Statistics Unit, The Institute of Cancer Research, London, UK; 4 Royal Bournemouth Hospital, Castle Lane East, Bournemouth, UK; 5 Royal Liverpool University Hospital, 200 London Road, Liverpool, UK; 6 Queen Elizabeth University Hospital Glasgow, Govan, UK; 7 Poole General Hospital, Longfleet Road, Dorset, UK; 8 University of Nottingham, Derby Rd., Nottingham, UK; 9 Breast Unit, Royal Marsden Hospital, London, UK

**Keywords:** Breast cancer, Oestrogen receptor, Aromatase inhibition, Resistance, Residual proliferation, Signatures

## Abstract

**Background:**

Endocrine therapy reduces breast cancer mortality by 40%, but resistance remains a major clinical problem. In this study, we sought to investigate the impact of aromatase inhibitor (AI) therapy on gene expression and identify gene modules representing key biological pathways that relate to early AI therapy resistance.

**Methods:**

Global gene expression was measured on pairs of core-cut biopsies taken at baseline and at surgery from 254 patients with ER-positive primary breast cancer randomised to receive 2-week presurgical AI (*n* = 198) or no presurgical treatment (control *n* = 56) from the POETIC trial. Data from the AI group was adjusted to eliminate artefactual process-related changes identified in the control group. The response was assessed by changes in the proliferation marker, Ki67.

**Results:**

High baseline *ESR1* expression associated with better AI response in HER2+ tumours but not HER2− tumours. In HER2− tumours, baseline expression of 48 genes associated with poor antiproliferative response (*p* < 0.005) including *PERP* and *YWHAQ*, the two most significant, and the transcription co-regulators (*SAP130*, *HDAC4*, and *NCOA7*) which were among the top 16 most significant. Baseline gene signature scores measuring cell proliferation, growth factor signalling (*ERBB2-GS*, RET/*GDNF-GS*, and *IGF-1-GS*), and immune activity (*STAT1-GS*) were significantly higher in poor AI responders. Two weeks of AI caused downregulation of genes involved in cell proliferation and ER signalling, as expected. Signature scores of E2F activation and TP53 dysfunction after 2-week AI were associated with poor AI response in both HER2− and HER2+ patients.

**Conclusions:**

There is a high degree of heterogeneity in adaptive mechanisms after as little as 2-week AI therapy; however, all appear to converge on cell cycle regulation. Our data support the evaluation of whether an E2F signatures after short-term exposure to AI may identify those patients most likely to benefit from the early addition of CDK4/6 inhibitors.

**Trial registration:**

ISRCTN, ISRCTN63882543, registered on 18 December 2007.

## Background

Breast cancer (BC) is the most common malignancy in women worldwide [1]. Over 80% [2] of primary BCs express oestrogen receptor (ER) alpha. While tamoxifen is an effective agent for reducing recurrence and death from BC, its effectiveness is impeded by its partial agonist activity. Aromatase inhibitors (AIs) show greater efficacy than tamoxifen. They reduce BC mortality by c.40% and have become the preferred first-line agent in postmenopausal women [3–5]. While treatment with an AI is sufficient to control the disease in many patients, for others, additional treatment to target resistance pathways is necessary, but identifying the mechanisms of resistance is mandatory to optimise this strategy.

Identifying relevant mechanisms of resistance in individual patients presenting with ER+ primary disease and treated post-surgically with adjuvant AI is prohibitively difficult, because patients are clinically disease-free after surgery and the absence of recurrence may relate to the absence of subclinical micrometastases or to disease control by the AI. In contrast, in the presurgical setting, gene expression in an individual tumour may be assessed in relation to validated markers of response in the same tumour. Multiple clinical trials provide strong evidence to support change in the expression of the nuclear proliferation marker, Ki67, after only 2-week treatment with an endocrine agent to be a valid predictor of the long-term benefit from adjuvant endocrine therapy and to be a better predictor of such benefit than clinical response [6–9]. In addition, the residual level of Ki67 after short exposure to endocrine therapy provides better prognostic information than pre-treatment Ki67 [10]. Thus, change in Ki67 can be used to measure a tumour’s response to AI and to study the mechanisms underpinning this, while residual level of Ki67 after short-term AI may be used to identify patients whose tumours retain significant proliferative drive, who are thereby at high-risk of recurrence and merit additional treatment. Identifying the molecular pathways associated with the residual Ki67 may allow such additional treatment to be targeted at relevant resistance pathway(s).

While a small number of presurgical studies have the potential to identify pathways associated with response and early resistance in ER+ patient populations, including some by our group [11, 12], most reports have lacked adequate patient numbers to allow the identification of effects restricted to subgroups of patients. In addition, and importantly, previous reports have not included controls that can identify artefacts that result from the experimental design of pre-surgical studies; we have recently reported that the changes in gene expression of the greatest magnitude in AI-treated patients in a short-term presurgical study are entirely artefactual. This makes the inclusion of a control set of tumours critical for eliminating these artefacts [13]. In the following analyses, we have utilised a study design that avoids these limitations by accessing samples from the PeriOperative Endocrine Therapy-Individualising Care (POETIC, CRUK/07/015) trial [14]. The inclusion of the no-treatment group in POETIC allowed us to adjust our observation in order to eliminate the impact of pre-analytical artefacts.

The POETIC trial, randomised postmenopausal women with primary ER+ BC 2:1 receive perioperative AI (2 weeks pre- + 2 weeks post-surgery, termed AI-treated) or no perioperative treatment (termed control). We report analyses from the cohort of 254 (AI-treated = 198; control = 56) patients from whom samples in RNA-later were available and provided high-quality genome-wide expression data. This is the largest presurgical study of the mechanisms of response and resistance to AIs to date and has sufficient numbers for separate analyses of HER2− and HER2+ subsets (i) to determine the associations between the baseline expression of individual genes or biological pathways with the change in Ki67 and the residual on-treatment Ki67 and (ii) to investigate the early impact of AI on gene expression and gene signatures.

## Methods

### Detailed methods are described within the STAR file (Additional file 1)

#### Patients and samples

**Fig. 1:**
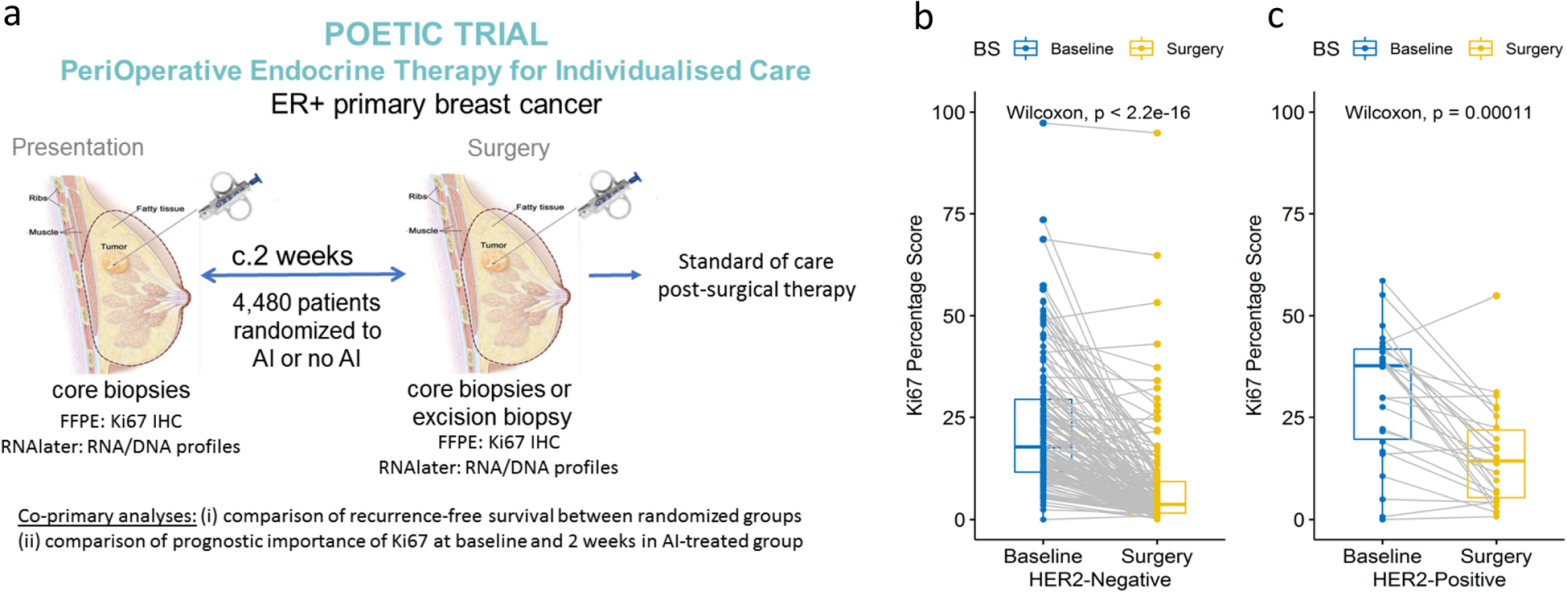
**a** POETIC schema, study design POETIC Trial PeriOperative Endocrine Therapy for Individualised Care. **b** Individual Ki67 changes in HER2− (*n* = 159) AI-treated groups. **c** Individual Ki67 changes in HER2+ (*n* = 26) AI-treated groups. The boxes indicate the median and interquartile ranges

The patients studied were a subpopulation of the POETIC (PeriOperative Endocrine-Therapy for Individualised Care) [14] study. The study design is illustrated in Fig. 1a.

#### RNA extraction

Total RNA was extracted using miRNeasy (Qiagen, Sussex, UK). RNA quality was checked using an Agilent Bioanalyser (Santa Clara, CA, USA), as previously described [15].

#### Ethics statement

Ethical approval for POETIC (Trial Number CRUK/07/015) was provided by NRES Committee London–South-East. All patients consented to molecular analysis of their samples for research purposes.

#### Gene expression analysis and data pre-processing

RNA amplification, labelling, and hybridization on HumanHT-12_V4 expression BeadChips (Illumina, San Diego, CA, USA) were performed, according to the manufacturer’s instructions. The raw data was extracted using GenomeStudio Software and was processed in R using lumi package (http://www.bioconductor.org) (Additional file 1).

#### Elimination of gene expression changes in the control group

To correct for potential artefactual changes in the gene expression that resulted from study procedures (10), the 2-week changes in the expression resulting from AI treatment were estimated for each gene by comparing the expression changes (log_2_^(Surgery/Baseline)^) in the AI-treated tumours and the expression changes (log_2_^(Surgery/Baseline)^) of the un-treated tumours. The relative (corrected) gene expression level in a given sample was calculated by subtracting the mean expression for the gene in the control samples from the expression of the given gene in the AI-treated tumour. All data shown that relate to either on-treatment expression/signature score or changes in the expression/signature score were corrected in this manner.

#### Biomarker analyses

Ki67% staining on formalin-fixed samples was carried out using anti-MIB-1 (M7240, DAKO UK), as previously described (7). HER2 status was measured locally using immunohistochemistry (IHC) and/or in situ hybridisation [16].

#### Published gene signatures

We determined the association of gene signatures representative of various biological processes for their association with the antiproliferative response to AI. In some cases, these signatures have been suggested as associated with resistance to endocrine therapy, and the work here can be considered an assessment of the validity of those findings: *Inflammatory-GS* [11], *STAT1-GS* [12], *IGF1-GS* [12], *RBloss-GS* and *DiLeoRBloss-GS* [17, 18], *E2Factivation-GS* [19], *E2F4-GS* [20], *TP53-GS* [21], and *GDNF-GS* [22]. For other signatures, our analyses were exploratory, and positive findings would need further validation. Many of the signatures have a predominance of known proliferation-associated genes (PAGs) that obscure the likely relationship with the signalling pathways per se; therefore, we conducted analyses that included and excluded PAGs from the respective signatures, as previously described [12] (Additional file 2: Table S1).

#### Immune or stromal score estimation

To allow comparison of the extent of immune or stromal admixture between samples, we used ESTIMATE [23].

#### Statistical analysis

Unpaired *t* tests were used to compare the mean changes in the gene expression (log_2_^[Surgery/Baseline]^) of tumours in the treated vs the control group using BRB-Array Tools (https://brb.nci.nih.gov/BRB-ArrayTools/). The Ingenuity Pathways Analysis (IPA) was conducted on the lists of genes that associated with the change in Ki67, or residual Ki67, or were differentially expressed to identify over-represented pathways. For individual pathways, the Benjamini-Hochberg procedure was used to calculate the false discovery rate (FDR) in order to adjust for multiple testing; the association between the two groups was considered to be statistically significant when *p* value < 0.005; the difference between the two sets of data was considered to be statistically significant when *p* value < 0.001. Reported *p* values are two-sided.

#### Endpoints

Four endpoints were used in this study: (i) change in Ki67 between baseline and 2 weeks as a continuous variable and (ii) responder or non-responder, defined as a reduction of > 60% or < 60%, respectively [24]; (iii) residual Ki67 as a continuous variable, and (iv) presence or absence of complete cell cycle arrest (CCCA or noCCCA), i.e. residual Ki67 < 2.7% or > 2.7%, respectively [25]. Each of the endpoints provides different information: (i) and (ii) reflect the antiproliferative response to AI treatment which relates to the benefit from the treatment, and endpoints (iii) and (iv) relate to the residual risk after AI therapy as described in a reference endpoints’ table (Additional file 2: Table S2). Patients with a baseline Ki67 value < 5% were excluded from (i) and (ii) because low pretreatment values can lead to highly aberrant estimates of proportional change.

## Results

### Patient demographics and changes in Ki67

There were 198 AI-treated patients with a baseline gene expression profile and paired Ki67 values (Additional file 8). Of these, 157 also had a gene expression profile at surgery. There were 56 controls with a gene expression profile at both baseline and surgery. Reasons for samples being excluded are shown in the consort diagram (Additional file 3: Figure S1). The demographics of the AI-treated patients are shown in Additional file 2: Table S3. Of the tumours, 81% were ductal and 61% were histologic grade 2. At surgery, 66% had a tumour diameter between 2 and 5 cm. All tumours were ER+, except 1 case which was found to be ER-negative after all analyses had been completed. Data on HER2 status, the individual changes in Ki67, and categorisation into responders or non-responders is shown in Additional file 2: Table S4.

Twenty-six (13.1%) of the AI-treated tumours and 8 (14.3%) of the control tumours were HER2+. Major between-patient heterogeneity in Ki67 change was evident in both the HER2− and HER2+ AI-treated groups, but there was significantly greater geometric mean suppression of Ki67 in the HER2− compared to the HER2+ cases (77.7% and 50.0%, respectively; *p* = 2.72E−04) (Fig. 1b, c). One hundred thirteen of 155 (72.9%) of the HER2− cases (with baseline Ki67 > 5%) were classed as good responders, compared with 9/23 (39.1%) HER2+ cases (Fisher’s exact test *p* = 2.90E−03). Furthermore, a higher proportion, 40.0% (66/161), of HER2− cases reached CCCA compared with 11.5% (3/26) of the HER2+ cases (Fisher’s exact test *p* = 4.00E−03) (Additional file 2: Table S5 a,b,c). This observation confirms previous studies indicating that the antiproliferative response to AIs is impeded in HER2+ tumours [26, 27]. As a consequence, all further analyses were conducted separately for HER2− and HER2+ subgroups.

## HER2-negative tumours

### Predictors of de novo antiproliferative response to AI

#### Association of individual genes and gene signatures with change in Ki67

Baseline expression of 123 genes correlated with the 2-week change in Ki67 with *p* value < 0.005 (Additional file 4: Figure S2; Additional file 2: Table S6). Of note, because the change is a reduction in Ki67, correlations with good response are negatively signed. High expression of 75 genes was associated with better response and 48 genes with poorer response. These 2 sets of genes segregated as the 2 major arms when the 123 genes were subjected to hierarchical clustering. The 6 genes with the strongest correlations were all genes associated with better response, but even for these, the absolute *r* values were all < 0.40 (Table 1; Additional file 2: Table S6). No further distinct groupings were apparent in the heatmap other than a tendency for non-luminal subtypes to show poorer Ki67 suppression.

**Table 1:** Genes whose baseline expression significantly correlated with the change in Ki67 (*p* < 0.005) based on 155 HER2− of the 178 AI-treated samples

Gene symbol	Entrez gene name	Correlate with change in Ki67			Correlate with residual Ki67		
		Rank by correlation coefficient (rho)	Rho	Parametric *p* value	Ranking by rho	Rho	Parametric *p* value
ESR1		NA	− 0.11	1.70E−01	NA	− 0.16	5.30E−02
Associated with good antiproliferative response							
ACADVL	Acyl-CoA dehydrogenase very long	1	− 0.355	6.90E−06	1	− 0.419	1.00E−07
TRABD	TraB domain containing	2	− 0.333	2.51E−05	208	− 0.241	2.58E−03
CCND1	Cyclin D1	3	− 0.328	3.34E−05	294	− 0.226	4.69E−03
TRIP6	Thyroid hormone receptor interactor 6	4	− 0.299	1.71E−04	58	− 0.286	3.18E−04
CTTN	Cortactin	5	− 0.297	1.88E−04	NA	NA	> 5.00E−3
MRPL23	Mitochondrial ribosomal protein L23	6	− 0.295	2.04E−04	277	− 0.228	4.44E−03
CCNI	Cyclin I	7	− 0.289	2.81E−04	89	− 0.271	6.79E−04
EPHX2	Epoxide hydrolase 2	7	− 0.289	2.82E−04	8	− 0.356	6.30E−06
IMPDH2	Inosine monophosphate	9	− 0.288	3.00E−04	114	− 0.264	9.45E−04
ARHGEF6	Rac/Cdc42 guanine nucleotide exchange factor 6	10	− 0.283	3.70E−04	NA	NA	> 5.00E−3
ACY1	Aminoacylase 1	10	− 0.283	3.73E−04	86	− 0.272	6.53E−04
EIF3G	Eukaryotic translation initiation factor 3 subunit G	12	− 0.282	3.91E−04	122	− 0.261	1.08E−03
GLI3	GLI family zinc finger 3	12	− 0.282	3.97E−04	155	− 0.252	1.59E−03
TTC17	Tetratricopeptide repeat domain 17	14	− 0.279	4.70E−04	6	− 0.366	3.30E−06
SCUBE2	Signal peptide, CUB domain and EGF-like domain containing 2	15	− 0.276	5.24E−04	3	− 0.393	5.00E−07
MRPS27	Mitochondrial ribosomal protein S27	16	− 0.274	5.84E−04	245	− 0.236	3.23E−03
Associated with poor antiproliferative response							
PERP	PERP, TP53 apoptosis effector	1	0.291	2.53E−04	161	0.273	6.09E−04
YWHAQ	Tyrosine 3-monooxygenase/tryptophan 5-monooxygenase activation protein theta	2	0.29	2.63E−04	86	0.328	3.47E−05
SNIP1	Smad nuclear interacting protein 1	3	0.275	5.47E−04	210	0.26	1.15E−03
PNO1	Partner of NOB1 homologue	4	0.274	5.74E−04	214	0.258	1.21E−03
SEC22B	SEC22 homologue B, vesicle trafficking protein (gene/pseudogene)	5	0.268	7.64E−04	NA	NA	> 5.00E−3
GOLT1B	Golgi transport 1B	5	0.268	7.92E−04	87	0.326	3.82E−05
SAP130	Sin3A-associated protein 130	7	0.264	9.56E−04	285	0.24	2.65E−03
GPKOW	G-patch domain and KOW motifs	8	0.261	1.08E−03	135	0.289	2.78E−04
NUS1	NUS1 dehydrodolichyl diphosphate synthase subunit	9	0.259	1.19E−03	187	0.266	8.67E−04
PLCB1	Phospholipase C beta 1	10	0.257	1.28E−03	NA	NA	> 5.00E−3
MBIP	MAP3K12 binding inhibitory protein 1	11	0.256	1.34E−03	NA	NA	> 5.00E−3
VCAM1	Vascular cell adhesion molecule 1	12	0.254	1.50E−03	132	0.29	2.62E−04
DNAJC8	DnaJ heat shock protein family (Hsp40) member C8	13	0.25	1.79E−03	NA	NA	> 5.00E−3
HENMT1 (C1orf59H)	EN methyltransferase 1	14	0.249	1.87E−03	201	0.261	1.08E−03
MRPL50	Mitochondrial ribosomal protein L50	15	0.246	2.07E−03	NA	NA	> 5.00E−3
ODF2	Outer dense fibre of sperm tails 2	16	0.245	2.15E−03	NA	NA	> 5.00E−3
PIGA	Phosphatidylinositol glycan anchor biosynthesis class A	16	0.245	2.16E−03	209	0.26	1.13E−03
HDAC4	Histone deacetylase 4	16	0.245	2.19E−03	311	0.236	3.21E−03
NCOA7	Nuclear receptor coactivator 7	16	0.245	2.21E−03	NA	NA	> 5.00E−3

The genes of top rank 16 that associated with good antiproliferative response and the genes of top rank 16 that associated with poor antiproliferative response, plus *ESR1*. All the 123 genes showing a significant correlation with the change in Ki67 are shown in Additional file 2: Table S5

Among the 48 genes whose high expression associated with poorer response, *PERP* (a TP53 apoptosis effector) and *YWHAQ* (tyrosine 3-monooxygenase/tryptophan 5-monooxygenase activation protein) were the top 2 best correlated genes (*r* = 0.291 and 0.290, respectively), while 3 transcription co-regulators, *SAP130*, *HDAC4*, and *NCOA7*, were among the top 16 most correlated with poor Ki67 repression (Table 1).

The most highly correlated of the genes associated with better response was *ACADVL* which is related to fatty acid degradation [28]. *CCND1* and *SCUBE2* which are known to be associated with better response to endocrine therapy [29, 30] were among the top 16 best correlated with good suppression of Ki67. *ESR1* expression was not correlated with the change in Ki67 after 2 weeks of AI therapy (Table 1; Additional file 5: Figure S3a).

Pathway analysis of the 123 genes identified HIPPO signalling as the most significantly over-represented pathway together with others directly or indirectly related to cell cycle regulation including p53 and p70S6K signalling (Additional file 6: Figure S4).

Of the pre-selected baseline signature scores, only proliferation-based modules (*Gene70-GS*, *GGI-GS*, *AURKA-GS*, *CIN70-GS*) and *Rbloss-GS* were significantly correlated with poor Ki67 response and these only weakly so (*r* = 0.243 to *r* = 0.161, all *p* < 0.05). *WntTarget34-GS* score was significantly correlated with good response, while *TP53-GS* score (signature associated with functional TP53) and several previously defined oestrogen signalling signatures approached significance (Additional file 7: Figure S5a; Additional file 8: Table S18A).

When the Ki67 changes were dichotomised to responders and non-responders, most of the baseline GSs whose score significantly associated with poor response were proliferation-based modules and *Rbloss* signatures, which was similar to the above. However, four additional GS that are not directly associated with proliferation but rather represent growth factor signalling pathways were significantly higher in non-responder tumours: *ERBB2-GS*, *IGF1-GS*, *STAT1-GS*, *GDNF-GS* (Table 2; Additional file 2: Table S7). Furthermore, five genes (*CCND1*, *EPHX2*, *TRIP6*, *IMPDH2*, and *ACADVL*) showed baseline expression that was significantly higher in AI responder tumours (*p* ≤ 1.5E−4);

**Table 2:** Unpaired *t* test of significance for the difference between the two group baseline gene expression means of (i) non-responders vs responders and (ii) noCCCAs vs CCCAs in HER2− group. The means of gene signatures that directly associated with proliferation and represent growth factor signalling pathways were significantly different between AI responder and non-responder tumours, and most of them were statistically different between CCCAs and noCCCAs

Module name	Reference	Non-responders vs responders				noCCCAs vs CCCAs			
		Mean (non-responders)	Mean (responders)	*t* test *p* value	FDR	Mean (no CCCAs)	Mean (CCCAs)	*t* test *p* value	FDR
ERBB2-GS.NoPAGs	Desmedt et al.	4.901	4.773	5.01E−03	3.78E−02	4.814	4.798	6.49E−01	6.49E−01
ERBB2-GS	Desmedt et al.	4.475	4.348	5.04E−03	3.78E−02	4.391	4.372	5.98E−01	6.35E−01
GGI-GS.NoPAGs	Sotiriou et al.	0.683	0.565	7.83E−03	3.78E−02	0.669	0.476	7.10E−07	3.02E−06
AURKA-GS	Desmedt et al.	2.323	2.217	1.10E−02	3.78E−02	2.311	2.139	4.82E−06	1.37E−05
IGF1-GS	Creighton et al.	-0.365	− 0.402	1.62E−02	3.78E−02	− 0.371	− 0.426	3.95E−04	8.39E−04
Immune.2.STAT1.NoPAGs	Desmedt et al.	7.720	7.526	1.83E−02	3.78E−02	7.608	7.526	2.27E−01	3.22E−01
STAT1-GS	Desmedt et al.	7.819	7.623	1.84E−02	3.78E−02	7.706	7.623	2.23E−01	3.22E−01
Gene70-GS	van ‘t Veer et al.	3.029	2.928	2.54E−02	3.78E−02	3.019	2.846	2.19E−06	7.45E−06
GDNF-GS.NoPAGs	Morandi et al.	1.586	1.475	2.61E−02	3.78E−02	1.520	1.474	2.74E−01	3.58E−01
removedPAGs	Gao et al.	0.555	0.507	2.65E−02	3.78E−02	0.552	0.467	7.46E−06	1.81E−05
WntTarget34-GS	VerhaeghCR2014TableS1	8.091	8.191	2.78E−02	3.78E−02	8.153	8.181	4.70E−01	5.33E−01
IGF1.NoPAGs	Creighton et al.	-0.603	− 0.636	2.91E−02	3.78E−02	− 0.608	− 0.657	1.09E−03	2.06E−03
GDNF-GS	Morandi et al.	1.699	1.591	3.08E−02	3.78E−02	1.634	1.594	3.31E−01	4.02E−01
E2Factivation-GS.NoPAGs	Miller et al.	9.119	9.029	3.12E−02	3.78E−02	9.091	8.996	7.83E−03	1.33E−02
JClinInvest2007RBloss-GS	Bosco et al.	7.961	7.802	3.44E−02	3.82E−02	7.960	7.654	2.83E−07	1.60E−06
GGI-GS	Sotiriou et al.	5.364	5.219	3.59E−02	3.82E−02	5.372	5.068	1.55E−07	1.60E−06
DiLeoRBloss-GS	Malorni et al.	7.771	7.600	4.21E−02	4.21E−02	7.781	7.423	2.29E−07	1.60E−06

#### Association of baseline gene expression and pre-selected signatures with 2-week residual Ki67

Baseline expression of 678 genes correlated with residual Ki67 after AI treatment. High expression of 376 genes was associated with high residual proliferation, and 302 genes were associated with low residual proliferation (Additional file 2: Table S8). Consistent with its association with good Ki67 suppression, *ACADVL* was the gene whose baseline expression was most strongly associated with low residual Ki67 (*r* = 0.419) and *SCUBE2* the third most strongly associated (Table 1). Interestingly, the baseline expression of *ACADVL* and *SCUBE2* was significantly correlated (*r* = 0.27, *p* = 0.0006). *ESR1* expression was not correlated with residual Ki67 (*r* = − 0.16, *p* = 5.3E−2; Table 1; Additional file 5: Figure S3b).

The gene whose baseline expression was most strongly associated with high residual Ki67 was *NEK2*, a kinase involved in centrosome separation and bipolar spindle formation (*r* = 0.478). *PTTG1* and the related *PTTG3P* were also among the top 5 most strongly correlated with residual Ki67 (*r* = 0.459 and 0.477, respectively). Both code for the members of the securin family that are homologues of yeast proteins that prevent separation of sister chromatid. Similarly, *CDCA5*, the third most strongly correlated gene, is also a regulator of sister chromatid cohesion, and all other genes strongly correlated at baseline with residual Ki67 are known to be associated with proliferation. Consistent with this, pathway analysis of the 678 genes showed p53, ATM, and EIF2 signalling pathways were among the most significantly over-represented (Additional file 2: Table S9), and of the pre-selected signatures, *TP53-GS* baseline score was the strongest inversely associated with residual Ki67 (*r* = − 0.46, *p* < 0.0001) (Additional file 7: Figure S5a; Additional file 8: Table S18A﻿). The inverse correlation relates to high *TP53-GS* score being associated positively with *TP53* wild-type status [21]. In contrast, baseline scores of *Gene70-GS*, *GGI-GS*, *Rbloss-GS*, *DiLeoRBloss-GS*, *CIN70-GS*, *E2F4activation-GS*, *E2FmotifCellCycleAssociated-GS*, *AURKA-GS*, *PTEN-GS*, and *E2Factivation-GS* score were positively correlated with residual Ki67 (all *r* ≥ 0.35, *p* < E−05).

As expected, higher baseline signature scores of *PIK3CA-GS* and modules measuring oestrogen signalling (*ERGs-GS*, *ESR1-1-GS*, *ESR1-2-GS*, *SET-GS*) were significantly associated with lower residual Ki67 (all *p* < 0.01). Higher *STAT1-GS* score was significantly but weakly correlated with higher residual Ki67 (*r* = 0.19, *p* = 1.57E−02) (Additional file 7: Figure S5a; Additional file 8: Table S18A).

#### Association of genes and pre-selected signatures with complete cell cycle arrest

**Fig. 2:**
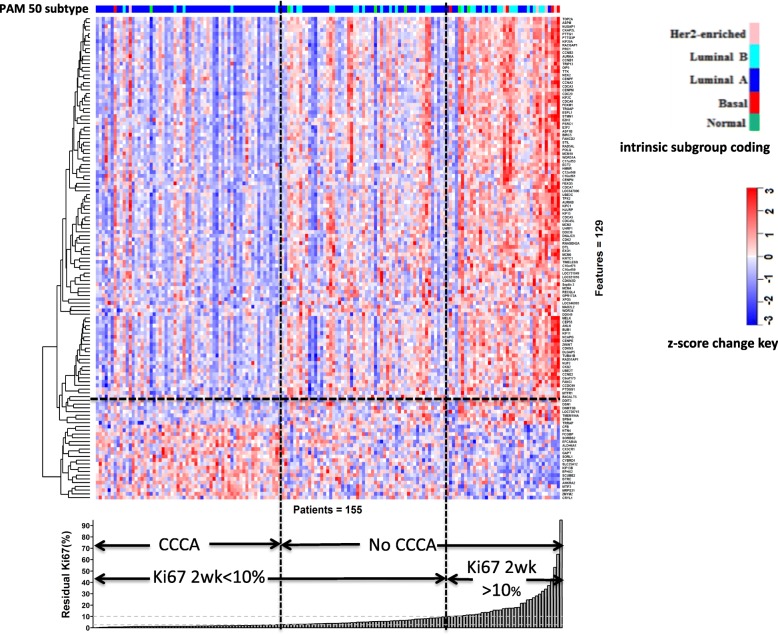
Heatmap (Pearson, complete) of 129 genes whose baseline expression is significantly different (*p* < 0.001) between CCCA and noCCCA based on 155 HER2− of the 178 AI-treated samples. The gene expression across 155 samples was centred and scaled. Red denotes the gene expression in a sample is greater than the mean, blue denotes less than the mean. The tumours are ordered according to the residual level of Ki67

The baseline gene expression of 129 genes was significantly different between tumours reaching CCCA and noCCCA. Of the 109 genes whose baseline gene expression was significantly higher in the noCCCA tumours, 71.5% were proliferation-associated (Fig. 2; Additional file 2: Table S10). Similar to the above analysis of associations with residual proliferation, high baseline expression of *PTTG1*, *PTTG3P*, *NEK2*, and *CDCA5* were prominent in being associated with noCCCA, but the most noticeable were *TOP2A* and *UBE2C*. High baseline *NEK2* expression was also associated with poor antiproliferative response (Additional file 4: Figure S2). Notably, 5 genes (*SCUBE2*, *FCGBP*, *EFCAB4A*, *EPHX2*, and *BTRC*) whose baseline expression was significantly higher in tumours that achieved CCCA (Fig. 2; Additional file 2: Table S10) were also associated with good antiproliferative response (Additional file 4: Figure S2; Additional file 2: Table S6). Furthermore, *ACADVL* baseline expression was higher in CCCA tumours (*p* = 0.001).

Of the pre-selected signatures, the baseline expression of *TP53-GS*, *PIK3CA-GS*, and *ERGs-GS* were significantly lower in noCCCA tumours. The lower *TP53-GS* score associated positively with dysfunctional TP53. In contrast, the expression of *GGI-GS*, *DiLeoRBloss-GS*, *Rbloss-GS*, *CIN70-GS*, *E2FmotifCellCycleAssociated-GS*, *Gene70-GS*, *E2F4activation-GS*, *AURKA-GS*, *PTEN-GS*, *E2Factivation-GS*, and *IGF1-GS* were significantly higher in the noCCCA tumours (all *p* < 0.0001) (Table 2; Additional file 2: Table S7).

One-dimensional clustering based on the relative baseline gene expression showed no distinct gene groups were apparent, and 5 of the 10 non-luminal tumours (excluding the normal-like) showed poorer than average Ki67 response to AI (Additional file 4: Figure S2). Of the 38 patients who had residual Ki67 (> 10%), 14 were from the original 33 (42%) luminal B tumours, 4 out of 5 (80%) were HER2-enriched, and 4 out of 5 (80%) were basal-like. Surprisingly, 13% of the original luminal A tumours (14 of the 106) were evident (Fig. 2).

### Effects of oestrogen deprivation by AI treatment on gene expression and associated pathways

**Fig. 3:**
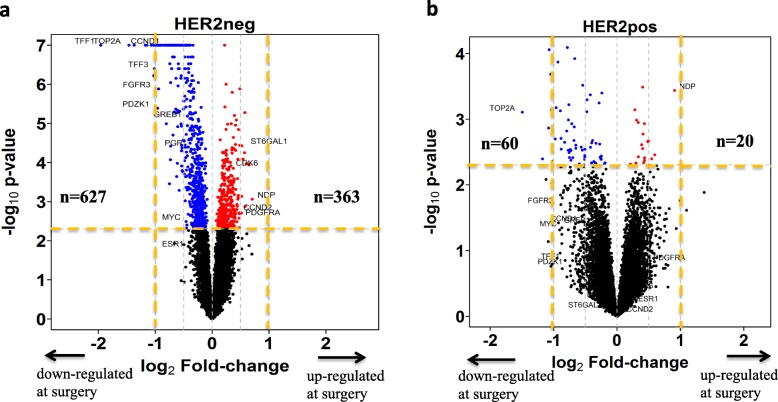
Volcano plot highlighting the genes that were identified differentially expressed (*p* < 0.005) after AI treatment. Based on the difference of the expression mean changes (log2^(Surgery/Baseline)^) of paired samples between AI-treated and control. **a** Nine hundred ninety genes (*n* = 363 upregulated, *n* = 627 downregulated) in HER2− tumours (902 annotated genes). Number of AI-treated pairs, *n* = 135; control pairs, *n* = 46. **b** Eighty genes (*n* = 20 upregulated, *n* = 60 downregulated) in HER2+ tumours (71 annotated genes). Number of AI-treated pairs, *n* = 22; control pairs, *n* = 8. The *p* values range from 1 to a limited minimum value of 1.0E−07 was shown on the *y*-axis in a scale of −log_10_^(*p* value)^

Oestrogen deprivation leads to profound effects on gene expression within 2 weeks. The expression of 902 genes was significantly changed: 560 downregulated and 342 upregulated (Fig. 3a; Additional file 2: Table S11). The most downregulated gene based on the amplitude of change was *TFF1*, followed by *UBE2C* and *TOP2A*, whose baseline expression was the most associated with noCCCA (both by > 60%). Similarly, *NEK2* the gene most associated with residual Ki67 as a continuous variable was the ninth most downregulated gene.

Forty-nine of the top 50 genes that showed the greatest change in expression were downregulated by AI. The large majority of these were either related to proliferation or regulated by oestrogen. *NDP* was the only upregulated gene based on the amplitude of change (FC = 1.63, *p* = 8.69E−04). *NDP* is a norrin cystine knot growth factor, which activates the canonical Wnt signalling pathway through the frizzled family of receptors (*FZD*). Of note, *FZD7*, frizzled class receptor 7 was also upregulated (FC = 1.23, *p* = 0.0002) [31]. Furthermore, *THRA*, thyroid hormone receptor, was highly upregulated by AI (Additional file 2: Table S12).

**Fig. 4:**
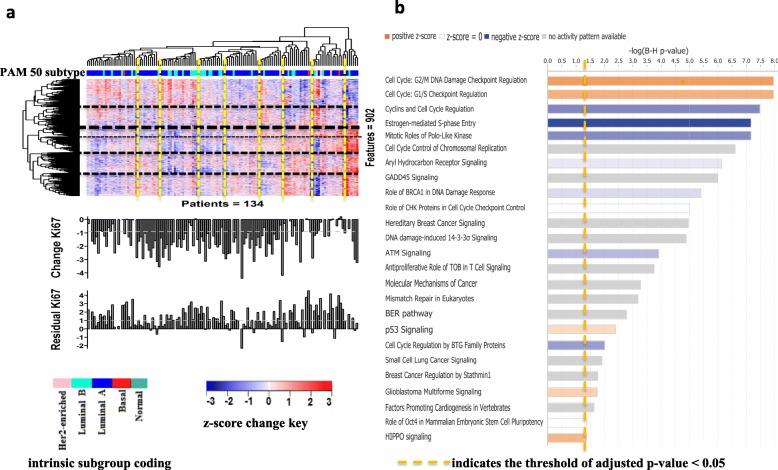
Unsupervised hierarchical clustering (Pearson, ward.D2) of 902 genes whose expression was significantly regulated after 2 weeks of treatment in HER2− tumours. And the overrepresented pathways (FDR < 5%) identified by pathway analysis (IPA). **a** The relative change in the gene expression across 134 HER2− tumours was standardised (centred and scaled). Red denotes the standardised *z*-score > 0, an increase in gene expression in a tumour after AI treatment compared to the average “relative changes” of the gene across all the 134 tumours; blue denotes the standardised *z*-score < 0, a decrease in gene expression in a tumour after AI treatment compared to the average “relative changes” of the gene across all the 134 tumours. **b** The 25 canonical pathways were significantly enriched (FDR < 5%). Positive *z*-score shown in orange colour specifies activated pathways; negative *z*-score shown in blue colour specifies inhibited pathways after AI treatment

The heterogeneity of the changes in the gene expression between patients, irrespective of the change in Ki67, is illustrated in Fig. 4a. A large number of distinct groups of tumours were apparent, but these groups show a little distinct relationship with intrinsic subgrouping or both the change in Ki67 and residual Ki67 levels.

Pathway analysis of the 902 genes that significantly changed with treatment revealed enrichment of 25 canonical pathways (adjusted *p* value < 0.05; Fig. 4b; Additional file 2: Table S13), the majority of which were proliferation-related. Cyclin-dependent kinases (*CDK1*, *2*, and *6*), *CHEK1*, cyclins (*CCNE1*, *2*; *CCND1*, *2*; and *CCNB1*, *2*), and transcription factors *E2F2* and *E2F5*, which were prominent in the majority of the 25 pathways, were also identified (Additional file 9: Figure S6).

Of particular note, *CDK6* and *CCND2* were significantly upregulated (*p* = 1.33E−04, *p* = 1.79E−03; Additional file 2: Table S12). In contrast to most of the cyclins and *CDKs*, *CCND2* is a cell cycle regulator whose activity is dependent on its binding to CDK4/6 in G1 phase. Phosphorylation of Rb (retinoblastoma) by CDK4/6-CCND2 uncouples Rb from *E2F* allowing transcription of essential S-phase genes. Inhibition of CDK4/6-*CCND2* in ER+ cells reduces cell proliferation and colony formation via a G1 cell cycle arrest [32]. The upregulation of *CCND2* and *CDK6* expression after AI therapy may be indicative of early tumour re-wiring that relates to residual proliferation.

Among the upregulated genes after AI treatment (Tables 3, 4, and 5; Additional file 2: Table S11), several (*SNAI2*, *TGFB3*, *TGFBR2*, *TWIST2*, *PDGFD*, *PDGFRA*, and *SMAD4*) are known to contribute to the loss of E-cadherin, a key mechanism in the stabilisation of the mesenchymal state that plays a role in the epithelial-mesenchymal transition (EMT) [33]. In addition, the increasing expression of *TGFBR2*, *ACVR1*, *TGFB3*, *SMAD4*, and *INHBB* are all linked to the activation of TGF-β signalling (*z*-score = 2.236) (Additional file 2: Table S13); the TGF-β signalling pathway has an established role in promoting EMT by downregulating E-cadherin via a number of transcription factors, such as Twist and Slug [34]. Finally, *FRMD6* and *YAP1*, members of the HIPPO pathway, were upregulated, while *LATS1*/*2*, known negative regulators of the pathway [35], were undetectable (Additional file 2: Table S11).

**Table 3:** Relative changes in the expression of gene signatures in response to the 2-week AI treatment of the HER2− and HER2+ tumours.

Module name	HER2-negative		HER2-positive		Significance of the difference between two changes in expression (HER2−vs HER2+)
	%Δ of geometric mean of intensities of a module of pre- and post-treatment	2-sided unadjusted *p* value of paired *t* test	Geometric mean of intensities of a module of pre- and post-treatment	Unadjusted *p* value of paired *t* test	
ESR1	− 23.06	1.54E−02	18.28	6.23E−01	9.18E−03
ERGs-GS	− 30.79	8.58E−14	− 21.96	2.85E−03	5.81E−02
DiLeoRBloss-GS	− 27.61	9.81E−10	− 30.64	1.41E−03	5.43E−01
JClinInvest2007RBloss-GS	− 26.11	5.47E−10	− 29.45	1.78E−03	4.72E−01
CIN70-GS	− 25.92	3.26E−10	− 28.69	1.81E−03	5.40E−01
E2F4activation-GS	− 25.26	4.54E−09	− 30.07	5.02E−03	3.13E−01
GGI-GS	− 23.67	1.78E−09	− 27.85	1.19E−03	3.40E−01
ERTarget27-GS	− 17.80	1.32E−15	− 11.41	3.32E−02	1.71E−02
E2FmotifCellCycleAssociated-GS	− 16.75	5.71E−09	− 21.72	2.00E−03	1.31E−01
E2Factivation-GS	− 14.45	2.25E−07	− 18.20	1.93E−02	2.62E−01
AURKA-GS	− 13.91	1.08E−07	− 15.45	3.94E−03	6.10E−01
Gene70-GS	− 9.37	1.97E−07	− 12.34	6.76E−03	1.98E−01
SET-GS	− 9.34	1.55E−05	− 2.08	5.69E−01	8.73E−03
PTEN-GS	− 7.55	1.44E−06	− 8.81	3.62E−02	5.38E−01
ESR1.2-GS	− 5.82	3.01E−04	− 0.46	8.49E−01	1.10E−02
ESR1.1-GS	− 5.40	2.99E−03	0.29	9.29E−01	1.50E−02
MYC-GS	− 4.36	1.98E−04	− 3.54	2.24E−01	6.02E−01
IGF1-GS	− 4.15	4.59E−04	− 4.07	7.76E−02	9.58E−01
VEGF-GS	− 3.85	3.33E−02	− 0.21	9.48E−01	1.09E−01
GDNF-GS	− 3.41	1.40E−01	− 5.23	4.25E−01	5.71E−01
obesity-GS	− 3.08	2.40E−02	− 1.72	5.64E−01	4.55E−01
E2F3-GS	− 2.89	5.01E−05	− 3.79	2.62E−02	3.45E−01
PI3K-GS	− 1.80	3.19E−03	− 3.11	6.69E−02	1.23E−01
SRC-GS	− 1.69	2.83E−01	− 1.62	6.46E−01	9.74E−01
AKT/mTOR-GS	− 1.62	4.57E−02	− 1.95	3.31E−01	7.51E−01
RAS-GS	− 1.47	1.93E−01	− 0.47	8.51E−01	5.04E−01
BetaCatenin-GS	− 1.28	3.83E−01	0.04	9.91E−01	5.07E−01
WntTarget34-GS	− 0.30	8.90E−01	− 2.56	6.50E−01	4.28E−01
ERBB2-GS	0.27	8.88E−01	− 0.88	8.60E−01	6.55E−01
PIK3CA-GS	0.39	7.20E−01	1.77	3.99E−01	3.42E−01
STAT1-GS	0.79	8.27E−01	− 7.73	2.98E−01	6.99E−02
CASP3-GS	1.37	5.57E−01	− 1.18	8.48E−01	3.82E−01
Bcell-GS	1.71	6.92E−01	− 7.38	3.51E−01	7.71E−02
MacTh1-GS	2.72	4.94E−01	0.20	9.79E−01	6.38E−01
Tcell-GS	2.83	5.38E−01	− 6.13	4.73E−01	1.12E−01
Inflammatory-GS	2.86	4.92E−01	− 4.80	5.15E−01	1.55E−01
Immune.1-GS	3.07	5.24E−01	− 5.34	6.10E−01	1.49E−01
MAPK-GS	3.21	6.59E−03	1.94	5.02E−01	4.22E−01
Stroma.2.PLAU-GS	6.57	4.65E−02	4.54	5.14E−01	6.34E−01
Stroma.1-GS	20.71	1.17E−02	21.87	1.61E−01	9.18E−01
TP53-GS	29.90	6.45E−10	29.38	5.59E−03	9.44E−01
PAGs	− 4.40	6.44E−06	− 6.23	2.06E−02	1.37E−01

Significant difference test of the changes between HER2− and HER2+ tumours. The increase of *TP53-GS* score after AI treatment is associated with the *TP53* wild-type status

**Table 4:** Spearman rank correlation of surgery *ESR1* expression/pre-selected gene signature scores and percentage of 2-week change in Ki67/residual Ki67 level in HER2− tumours

Surgery gene signature scores		HER2− (*n* = 135)					
		Change in Ki67 (*n* = 121)			Residual Ki67 (*n* = 124)		
Signature name	Reference	Rho	*p* value	FDR	Rho	*p* value	FDR
ESR1		− 0.19	3.42E−02	4.62E−02	− 0.17	6.53E−02	6.53E−02
TP53-GS	Coutant et al.	− 0.47	7.43E−08	6.50E−07	− 0.54	6.61E−11	3.57E−10
ERTarget27-GS	VerhaeghCR2014TableS2	− 0.26	3.96E−03	8.22E−03	− 0.25	5.25E−03	7.46E−03
SET-GS	Symmans et al.	− 0.25	5.38E−03	1.04E−02	− 0.3	7.82E−04	1.41E−03
ESR1.2-GS	Desmedt et al.	− 0.24	8.58E−03	1.32E−02	− 0.27	2.52E−03	4.00E−03
ESR1.1-GS	Mackay et al.	− 0.23	1.15E−02	1.63E−02	− 0.29	1.34E−03	2.26E−03
ERGs-GS	Dunbier et al.	− 0.09	3.56E−01	3.70E−01	− 0.21	1.96E−02	2.21E−02
PIK3CA-GS	Loi et al.	− 0.05	5.53E−01	5.53E−01	− 0.18	4.07E−02	4.23E−02
MacTh1-GS	Iglesia et al.	0.088	3.39E−01	3.66E−01	0.199	2.64E−02	2.85E−02
Inflammatory-GS	Dunbier et al.	0.116	2.05E−01	2.31E−01	0.22	1.43E−02	1.68E−02
GDNF-GS	Morandi et al.	0.116	2.04E−01	2.31E−01	0.254	4.40E−03	6.60E−03
obesity-GS	Creighton et al.	0.134	1.43E−01	1.76E−01	0.239	7.61E−03	9.34E−03
Immune.2.STAT1-GS	Desmedt et al.	0.158	8.26E−02	1.06E−01	0.247	5.64E−03	7.61E−03
E2F3-GS	Bild et al.	0.237	8.79E−03	1.32E−02	0.24	7.38E−03	9.34E−03
IGF1-GS	Creighton et al.	0.24	8.06E−03	1.32E−02	0.303	6.22E−04	1.20E−03
AKT/mTOR-GS	Majumder et al.	0.249	5.83E−03	1.05E−02	0.362	3.63E−05	7.54E−05
PTEN-GS	Saale at al.	0.286	1.49E−03	3.35E−03	0.392	6.68E−06	1.50E−05
PI3K-GS	Creighton 2010	0.291	1.21E−03	2.97E−03	0.413	1.92E−06	4.71E−06
AURKA-GS	Desmedt et al.	0.32	3.53E−04	9.53E−04	0.427	7.36E−07	1.99E−06
E2Factivation-GS	Miller et al.	0.323	3.08E−04	9.24E−04	0.432	5.39E−07	1.62E−06
E2FmotifCellCycleAssociated-GS	Miller et al.	0.402	4.79E−06	1.62E−05	0.454	1.22E−07	4.12E−07
Gene70-GS	van ‘t Veer et al.	0.431	8.22E−07	3.17E−06	0.546	5.64E−11	3.57E−10
CIN70-GS	Carter et al.	0.442	3.89E−07	1.75E−06	0.529	2.79E−10	1.08E−09
E2F4activation-GS	Guerrero-Zotano et al.	0.454	1.73E−07	9.34E−07	0.535	1.51E−10	6.80E−10
GGI-GS	Sotiriou et al.	0.462	9.63E−08	6.50E−07	0.571	4.52E−12	6.10E−11
JClinInvest2007RBloss-GS	Bosco et al.	0.491	1.12E−08	1.51E−07	0.545	5.89E−11	3.57E−10
DiLeoRBloss-GS	Malorni et al.	0.526	5.61E−10	1.51E−08	0.584	1.08E−12	2.92E−11

*TP53-GS* surgery score was the strongest inversely associated with the change in Ki67 and residual Ki67. The inverse correlation relates to high *TP53-GS* score being associated positively with TP53 wild-type status

**Table 5:** Spearman rank correlation of change in ESR1 expression/pre-selected gene signature scores and percentage of 2-week change in Ki67/residual Ki67 level in (i) HER2− tumours, (ii) HER2+ tumours, and (iii) significance of the difference between the two correlation coefficients (HER2− vs HER2+).

Change in gene signature scores		HER2− (*n* = 135)				HER2+ (*n* = 22)				Significance of the difference between two correlation coefficients (HER2− vs HER2+)	
		Change in Ki67 (*n* = 121)		Residual Ki67 (*n* = 124)		Change in Ki67 (*n* = 21)		Residual Ki67 (*n* = 22)			
Signature name	Reference	Rho	*p* value	Rho	*p* value	Rho	*p* value	Rho	*p* value	Change in Ki67	Residual Ki67
ESR1		− 0.08	3.58E−01	0.03	7.37E−01	0.41	6.70E−02	0.48	2.20E−02	3.69E−02	4.61E−02
E2F4activation-GS	Guerrero-Zotano et al.	0.38	2.12E−05	0.13	1.58E−01	0.42	5.84E−02	0.18	4.22E−01	8.47E−01	8.36E−01
DiLeoRBloss-GS	Malorni et al.	0.36	4.29E−05	0.09	3.12E−01	0.40	7.55E−02	0.23	2.94E−01	8.50E−01	5.60E−01
JClinInvest2007RBloss-GS	Bosco et al.	0.35	1.05E−04	0.08	3.87E−01	0.38	8.53E−02	0.22	3.31E−01	8.89E−01	5.62E−01
GGI-GS	Sotiriou et al.	0.34	1.30E−04	0.08	3.75E−01	0.41	6.28E−02	0.24	2.77E−01	7.42E−01	5.05E−01
CIN70-GS	Carter et al.	0.33	2.16E−04	0.09	3.26E−01	0.43	5.18E−02	0.23	3.04E−01	6.36E−01	5.60E−01
E2FmotifCellCycleAssociated-GS	Miller et al.	0.31	4.37E−04	0.04	6.91E−01	0.53	1.38E−02	0.09	6.84E−01	2.75E−01	8.39E−01
Gene70-GS	van ‘t Veer et al.	0.31	5.01E−04	0.05	5.90E−01	0.44	4.65E−02	0.14	5.46E−01	5.39E−01	7.13E−01
E2Factivation-GS	Miller et al.	0.29	1.49E−03	0.13	1.40E−01	0.42	5.92E−02	0.04	8.59E−01	5.46E−01	7.14E−01
ERGs-GS	Dunbier et al.	0.25	5.44E−03	0.28	1.96E−03	0.11	6.30E−01	0.49	2.07E−02	5.58E−01	3.15E−01
PTEN-GS	Saale at al.	0.23	1.16E−02	0.04	6.24E−01	0.46	3.43E−02	0.20	3.79E−01	2.87E−01	5.10E−01
PI3K-GS	Creighton 2010	0.23	1.31E−02	0.12	1.95E−01	0.50	1.99E−02	0.37	9.24E−02	2.02E−01	2.79E−01
AURKA-GS	Desmedt et al.	0.21	2.32E−02	0.01	8.70E−01	0.46	3.37E−02	0.38	7.72E−02	2.50E−01	1.15E−01
E2F3-GS	Bild et al.	0.20	3.20E−02	0.07	4.34E−01	0.43	5.18E−02	0.33	1.36E−01	2.98E−01	2.70E−01
WntTarget34-GS	VerhaeghCR2014TableS1	0.17	6.11E−02	0.16	6.99E−02	0.11	6.26E−01	− 0.09	6.91E−01	8.04E−01	3.09E−01
AKT/mTOR-GS	Majumder et al.	0.15	9.44E−02	0.14	1.20E−01	0.29	2.03E−01	0.25	2.64E−01	5.51E−01	6.43E−01
IGF1-GS	Creighton et al.	0.13	1.61E−01	− 0.05	5.80E−01	0.32	1.54E−01	0.42	5.41E−02	4.16E−01	4.41E−02
ERTarget27-GS	VerhaeghCR2014TableS2	0.12	1.86E−01	0.19	3.26E−02	0.29	1.97E−01	0.41	5.85E−02	4.72E−01	3.25E−01
MacTh1-GS	Iglesia et al.	0.10	2.57E−01	0.09	3.23E−01	− 0.17	4.51E−01	− 0.14	5.33E−01	2.71E−01	3.50E−01
CASP3-GS	Desmedt et al.	0.09	3.06E−01	0.12	2.00E−01	0.17	4.64E−01	− 0.08	7.32E−01	7.42E−01	4.17E−01
STAT1-GS	Desmedt et al.	0.07	4.50E−01	0.02	8.06E−01	0.11	6.30E−01	− 0.22	3.18E−01	8.70E−01	3.24E−01
GDNF-GS	Morandi et al.	0.06	4.80E−01	0.04	6.99E−01	0.20	3.94E−01	0.03	8.91E−01	5.64E−01	9.68E−01
MYC-GS	Bild et al.	0.06	5.30E−01	− 0.10	2.60E−01	0.40	7.14E−02	0.43	4.41E−02	1.41E−01	2.34E−02
Inflammatory-GS	Dunbier et al.	0.06	5.34E−01	0.03	7.56E−01	− 0.19	3.97E−01	− 0.28	2.04E−01	3.07E−01	1.99E−01
Bcell-GS	Iglesia et al.	0.05	5.55E−01	0.02	7.97E−01	− 0.01	9.55E−01	− 0.54	9.42E−03	8.08E−01	1.16E−02
Tcell-GS	Iglesia et al.	0.05	5.61E−01	− 0.02	8.09E−01	− 0.41	6.46E−02	− 0.33	1.36E−01	4.95E−02	1.92E−01
obesity-GS	Creighton et al.	0.05	5.67E−01	− 0.12	1.91E−01	− 0.22	3.33E−01	0.23	3.06E−01	2.68E−01	1.51E−01
VEGF-GS	Desmedt et al.	0.03	7.41E−01	− 0.02	8.34E−01	0.46	3.43E−02	0.63	1.57E−03	5.87E−02	2.07E−03
Stroma.2.PLAU-GS	Desmedt et al.	0.02	8.14E−01	0.01	9.55E−01	− 0.21	3.51E−01	− 0.17	4.46E−01	3.46E−01	4.62E−01
BetaCatenin-GS	Bild et al.	0.02	8.50E−01	− 0.07	4.09E−01	− 0.02	9.29E−01	0.33	1.39E−01	8.71E−01	9.48E−02
Stroma.1-GS	Farmer at al.	0.00	9.71E−01	0.04	6.27E−01	− 0.20	3.88E−01	− 0.26	2.51E−01	4.12E−01	2.16E−01
MAPK-GS	Creighton et al.	− 0.01	9.24E−01	− 0.07	4.24E−01	− 0.27	2.36E−01	− 0.30	1.70E−01	2.80E−01	3.33E−01
Immune.1-GS	Teschendorff at al.	− 0.01	9.12E−01	− 0.03	7.16E−01	− 0.16	4.78E−01	0.03	8.79E−01	5.40E−01	8.08E−01
PIK3CA-GS	Loi et al.	− 0.01	9.00E−01	0.02	8.03E−01	− 0.23	3.22E−01	− 0.15	5.13E−01	3.64E−01	4.89E−01
SRC-GS	Bild et al.	− 0.03	7.43E−01	− 0.13	1.58E−01	− 0.06	7.97E−01	− 0.14	5.46E−01	9.03E−01	9.67E−01
ESR1.1-GS	Mackay et al.	− 0.05	6.16E−01	0.07	4.16E−01	0.31	1.71E−01	0.42	4.99E−02	1.34E−01	1.27E−01
SET-GS	Symmans et al	− 0.05	5.70E−01	0.09	3.21E−01	0.22	3.28E−01	0.45	3.36E−02	2.68E−01	1.11E−01
RAS-GS	Bild et al.	− 0.06	4.99E−01	− 0.13	1.39E−01	0.40	7.44E−02	0.11	6.29E−01	5.04E−02	3.29E−01
ESR1.2-GS	Desmedt et al.	− 0.10	2.81E−01	0.05	5.69E−01	0.29	1.97E−01	0.62	2.21E−03	1.07E−01	6.32E−03
ERBB2-GS	Desmedt et al.	− 0.24	9.08E−03	− 0.22	1.20E−02	− 0.04	8.80E−01	0.15	5.06E−01	4.08E−01	1.29E−01
TP53-GS	Coutant et al.	− 0.37	2.38E−05	− 0.12	1.82E−01	− 0.32	1.63E−01	− 0.32	1.43E−01	8.18E−01	3.93E−01
PAGs	Gao et al.	0.27	3.11E−03	0.07	4.68E−01	0.47	2.99E−02	0.11	6.12E−01	3.45E−01	8.70E−01

Change in *TP53-GS* score was the strongest inversely associated with the change in Ki67 after 2-week AI treatment. The inverse correlation relates to the increase of *TP53-GS* score being associated positively with TP53 wild-type status

We next assessed the dynamic changes in the pre-selected signature response to 2-week AI treatment. *ESR1* gene expression and ER-regulated/targeted genes (*ERG-GS*, *ERTarget27-GS*, and several proliferation-associated GSs were profoundly reduced by AI (%∆ of geometric mean > 10%)), but none to the same magnitude as the single IHC marker Ki67 (Table 3; Additional file 2: Table S5). Module scores of *Gene70-GS*, *SET-GS*, *MYC-GS*, *PTEN-GS*, and *IGF1-GS* were also all significantly suppressed but to a lesser degree. In contrast, the scores of *Stroma.1-GS* and *TP53-GS* had largely increased due to oestrogen deprivation. The increased *TP53-GS* score associated positively with TP53 wild-type status.

### Association of 2-week pre-selected gene signature scores with changes in Ki67 and residual Ki67

On-treatment gene expression may be at least as important a determinant of resistance to AI therapy and a potential target for additional treatment as pre-treatment gene expression. We therefore assessed the association of on-treatment scores of the pre-selected signatures with the change in Ki67 and residual Ki67 (Table 4; Additional file 10: Figure S7a; Additional file 8: Table S19A). Significant correlations were found with several of the signatures and residual Ki67, and most of these were also significant for change in Ki67. Those correlations significant for both endpoints were (i) the two RB loss signatures [17, 18], (ii) proliferation-related signatures (*GGI-GS*, *CIN70-GS*, *Gene70-GS*, *AURKA-GS*), (iii) modules measuring oestrogen signalling (*SET-GS*, *ESR1.1-GS*, *ESR1.2-GS*, *ERTarget27-GS*), (iv) *E2F* signatures [19, 20], and (v) *TP53-GS*, *PI3K-GS*, *PTEN-GS*, *AKT/mTOR-GS*, and *IGF1-GS*. Of note, while high on-treatment oestrogen signalling module scores associated with lower residual proliferation and better antiproliferative response, high *TP53-GS* score that reflects wild-type TP53 function showed the highest correlation.

We found no significant relationship between the change in Ki67 and immune response gene signatures including *Inflammatory-GS* and the immune and stromal scores estimated by ESTIMATE. However, high *STAT1-GS* treatment score showed a significant association with high residual Ki67 (*r* = 0.25, *p* = 5.64E−03), as did the *Inflammatory-GS* and *MacTh1-GS* (Table 4).

### Association of the change in pre-selected gene signature scores with changes in Ki67 and residual Ki67

Unsurprisingly, ten of the changes in signature scores that were significantly directly correlated with Ki67 change were proliferation-associated GSs. However, of particular note, reduction in the expression of the *ERGs-GS* was also directly associated with greater Ki67 suppression and low residual Ki67. In addition, increase in *ERBB2-GS* score was significantly associated with both greater Ki67 suppression and lower residual Ki67 after AI therapy, possibly as an immediate compensatory resistance mechanism (Table 5; Additional file 2: Table S14; Additional file 11: Figure S8a; Additional file 8: Table S20A). The change in *ESR1* expression was significantly associated with the change in all the modules measuring oestrogen signalling (*SET-GS*, *r* = 0.72; *ESR1-1-GS*, *r* = 0.69; *ESR1-2-GS*, *r* = 0.59; ERTarget27*-GS*, *r* = 0.39; *ERGs-GS*, *r* = 0.36; all *p* < 0.0001).

## HER2-positive tumours

Class comparison of the mean changes between the 26 AI-treated HER2+ tumours and 8 HER2+ control tumours identified 71 annotated genes, which were significantly changed by AI therapy (*n* = 19 upregulated, *n* = 52 downregulated). (Fig. 3b; Additional file 2: Table S15). Pathway analysis of the 71 genes identified 7 canonical pathways as being significantly enriched (adjusted *p* value< 0.05; Additional file 12: Figure S9). Activation of the top pathway, mitotic roles of Polo-like kinase, was indicated as being significantly reduced by oestrogen deprivation consistent with the partial reduction in Ki67 for almost all of the HER2+ tumours and with the changes in proliferation-related genes in the HER2− cohort.

To identify any significant differences between HER2+ and HER2− tumours in their molecular response to AIs, we compared the AI-induced gene changes between the two groups (Additional file 2: Table S12). Seven of the 10 top downregulated genes in the HER2+ group were in the top 13 downregulated genes in HER2− tumours. The top upregulated gene *NDP* in the HER2− group was also the top upregulated in HER2+ tumours. Proliferation-associated and cell cycle genes were suppressed to a similar extent in both cohorts despite the difference in Ki67 suppression.

The classical oestrogen-regulated genes were suppressed to a significantly lesser extent by AI treatment in the HER2+ tumours, for example, downregulation of *TFF1*, *TFF3*, *CCND1*, and *PGR* was significantly less (*p*’s for difference = 0.0027, 0.0001, 0.035, and 0.0034, respectively). In contrast to the decrease in *ESR1* levels seen in the HER2− tumours, in HER2+ tumours, *ESR1* gene expression was not significantly changed (*p* = 0.009 for the difference between the groups). The GSs that measure oestrogen signalling (*ERTarget27-GS*, *SET-GS*, *ESR1.2-GS*, *ESR1.1-GS*) were also significantly less suppressed by AI in HER2+ tumours (Table 3). Again, in contrast with HER2− tumours, *ESR1* expression was significantly correlated with the change in Ki67 (*r* = − 0.61, *p* = 2.57E−03) being among the 25 genes whose baseline expression correlated with better Ki67 response (Additional file 5: Figure S3c; Additional file 2: Table S16). *ESR1* was among the 54 genes whose high baseline expression correlated with low residual proliferation in HER2+ tumours (*r* = − 0.62, *p* = 2.19E−03) while there were no such significant relationships with *ESR1* in the HER2− group (Additional file 5: Figure S3d; Additional file 2: Table S17).

### Association of genes and pre-selected signatures in HER2+ tumours

Analysis of the pre-selected signatures in the HER2+ cohort showed similar results to those observed in the HER2− tumours (Additional file 7: Figure S5b, Additional file 10: Figure S7b, Additional file 11: Figure S8b; Additional file 8: Table S18B, S19B, S20B). In those cases where there were differences between the relationships in HER2+ and HER2−, the *p* values were only ever moderately significant. Given the size of the HER2+ group and the multiple tests conducted, we did not pursue these further.

## Discussion

AIs are well-established as the most effective and therefore most frequently used endocrine agents for treating ER+ BC in postmenopausal women [5]. Despite the efficacy of AIs, many patients recur with either de novo or acquired AI-resistant disease. Molecular characterisation of the resistance phenotype(s) is critical for enhanced control of the disease. In this study, we report the largest sample set describing the genome-wide transcriptional and related antiproliferative effects of AIs. In addition, for the first time, we have been able to correct for artefactual transcriptional changes that occurred in the control group in the absence of any treatment. We have described those artefactual changes in detail elsewhere and discussed the likely causes [13, 15]. Most importantly, had we been unable to correct for them; the most significantly and most quantitatively changed genes in the AI-treated group would have been accepted in error as due to AI treatment while they were in fact entirely related to tissue processing [13]. Our analyses applied rigorous statistical methods using Benjamini-Hochberg procedure where appropriate to calculate the FDR in order to adjust for multiple testing.

Our assessment of the biologic response/resistance of the tumours to AI was based on Ki67. It is important to appreciate the significance of the different endpoints and their validity for that purpose. Proportional or percentage change in Ki67 has been validated as reflecting the degree of benefit (or response) to AI [6–9] and is the relevant parameter for considering mechanisms of response/resistance to AI therapy. The 2-week (residual) value of Ki67 is determined in part by the proportional response to the AI but also by the pretreatment value; the value correlates with the residual risk of recurrence on the endocrine therapy [10] and is relevant as a marker of the value/need to apply additional adjuvant therapy, irrespective of whether or not there has been a good or poor proportional antiproliferative response to the AI. Continued or altered (rewired) signalling in the residual tissue may be more relevant to the targeting of the additional agents than baseline expression. It should be noted that while our sample set was drawn from the POETIC trial, we make no claims for it being a representative subset (although it may be). Rather, the design of POETIC and availability of the set of RNAlater-stored samples provided the opportunity for us to undertake the molecular analyses described; the relevance of our observations to ER+ breast cancer in general may be considered by reference to the demographics of this subset.

In the HER2− group, we confirmed that high baseline signature scores of *IGF1-GS*, *STAT1-GS*, and *GDNF-GS* were associated with poor antiproliferative response when Ki67 change was dichotomised [12, 22]. Although we validated *STAT1-GS*, which represents features of immune activity as being associated with AI resistance, there was no significant association between Inflammatory-GS and the change in Ki67. This is somewhat in contrast to our previous report in a smaller mixed HER2−/HER2+ set in which both these signatures were predictive of poor AI response of tumours [11, 12]. The significantly high baseline *ERBB2-GS* in AI non-responders suggests tumours with high HER2 signalling activity even in HER2− tumours were predictive of poor response. This is consistent with the observation of poor response to letrozole alone and improved outcome with added lapatinib in the HER2-enriched subtype of HER2− metastatic BC [36]. Further assessment of the interaction between this subtype and response to endocrine therapy is now underway in the much larger formalin-fixed set of tissues from POETIC. The strong correlations between several baseline signature scores and the residual Ki67 confirmed the high proliferation (*AURKA-GS*, *PTEN-GS*, *Gene70-GS*, *GGI-GS*, *CIN70-GS*), RB-loss (*Rbloss-GS*, *DiLeoRBloss-GS*), high E2F activation (*E2F4activation-GS*, *E2Factivation-GS*), and TP53 dysfunction (*TP53-GS*) were associated with high-oestrogen independent residual proliferation irrespective of whether the tumour showed an antiproliferative response to AI [12, 17–21].

We and others have previously described that HER2 positivity impedes the antiproliferative effect of endocrine therapy [26, 27]. The resultant major difference in the changes in Ki67 suppression seen here between the HER2+ and HER2− group led us to consider the HER2 subgroups separately. This allowed us to describe the substantial differences in oestrogen signalling that occurred between them. In HER2+ but not HER2− tumours, baseline *ESR1* expression was significantly correlated with the change in Ki67 levels, and while those *ESR1* levels were suppressed in HER2− tumours, they were not significantly changed in HER2+ tumours; expression of oestrogen-regulated genes and ER-related gene modules was also changed less in HER2+ than in HER2− tumours. This difference could be explained by the decrease in oestrogen signalling in the HER2− tumours being in part dependent on the lower *ESR1* levels on-treatment and not just by the oestrogen deprivation with the AI. Alternatively, or in addition, the apparent persistent oestrogen signalling in HER2+ tumours might result from ligand-independent activation of ER by HER2. This highlights the complex crosstalk between HER2 and ER [37].

Assessment of the gene expression at baseline in the HER2− cohort to identify de novo biomarkers of resistance revealed a very marked heterogeneity between tumours with no new patterns of expression being associated with changes in Ki67. However, intrinsic subgrouping did reveal that luminal B and particularly the small number of non-luminal tumours showed less Ki67 suppression and greater residual Ki67 levels than luminal A tumours. This is somewhat in contrast to our report in a smaller earlier set of tumours in which the proportional change in Ki67 was found to be similar between luminal A and B tumours although the residual level of Ki67 was higher in the latter [11, 38].

Unsurprisingly, it is clear that proliferation and cell cycle-associated pathways dominated the gene signatures found to change with AI and also to be associated with residual Ki67. However, we also found that the baseline expression of several proliferation-related gene signatures was also related to the change in Ki67. This is consistent with the greater preponderance of luminal B and non-luminal tumours with a poor change in Ki67.

In contrast to the many cell cycle genes that were decreased in activity in parallel with the decrease in proliferation, *CCND2* and *CDK6* were increased. CDK4/6 inhibitors are now in widespread use in the treatment of ER+ metastatic BC and are in large clinical trials in primary BC, in each case in combination with endocrine therapy. It was also notable that the most prominent genes in the canonical pathways were *CDK2* and CCNEs which are critical to triggering the G1- to S-phase transition. As previously reported, on-treatment *E2F* signatures were among those most strongly associated with residual Ki67 [19, 39], and the *TP53-GS* that reflects wild-type TP53 function showed the highest correlation with lower residual Ki67. Assessment of the early impact by AIs on each of these factors may be relevant to the success or not of CDK4/6 inhibition when combined with an AI. This argues for an initial treatment with an AI before the introduction of the CDK4/6 inhibitor. We are pursuing this concept in the design of a new clinical trial of the adjuvant use of CDK4/6 inhibition in high-risk ER+ disease (POETIC-A).

A particularly novel finding was that *ACADVL* baseline expression was the best predictor of both decrease in Ki67 and of low residual Ki67, and its expression was significantly higher in responder and CCCA tumours. In silico analysis of the BC dataset reveals that the lower baseline expression of *ACADVL* was associated with poor relapse-free survival in ER+ patients [40]. The gene encodes a very long chain-specific acyl-CoA dehydrogenase, mitochondrial (VLCAD) enzyme, a key enzyme of the mitochondrial fatty acid β-oxidation (FAO) pathway. A recent study [41] revealed that VLCAD interacts with the BH3 domain of MCL-1 via a non-canonical mechanism, which is associated with chemoresistance in human cancer and merits further study.

Two other novel findings were the high baseline expression of (i) *PERP* and *YWHAQ* as the most significantly associated with poor AI response, and (ii) *NEK2* was most strongly associated with high residual proliferation. In silico analysis of the BC dataset reveals that high baseline expression of *YWHAQ* and *NEK2* have been reported to be associated with poor relapse-free survival in an ER+/HER2− setting for the patients receiving endocrine therapy and no chemotherapy [40]. Furthermore, the expression of *YWHAQ* and *NEK2* was significantly higher in luminal B compared to luminal A tumours in TCGA ER+/HER2− tumours [42]. Together, these findings suggest that the poor prognosis associated with these two genes may be at least partly due to an association with endocrine resistance. *PERP*, an apoptosis-associated target of p53, is a novel member of the PMP-22 family. A recent study [43] revealed that *PERP* is lost in more aggressive sparsely granulated human growth hormone pituitary tumours, and its loss and associated desmosomal instability may be an early driver of tumour progression. However, its significant association with poor antiproliferative response to AIs in ER+/HER2− tumours has not been previously reported and requires validation prior to further study.

## Conclusions

It is clear from the above that our work identifies the possible involvement of multiple pathways in de novo resistance to AIs, some but not all of which have previously been described. However, there are other pathways whose baseline activity is unrelated to resistance but whose expression is modified or rewired within the first 2 weeks and at that stage is related to residual proliferation.

While the number of cases described is the largest reported to date and is sufficient to identify the possible involvement of each of the pathways described, their relative importance will require assessment in a yet larger population.

Overall, we conclude that there is a high degree of heterogeneity between tumours in their adaptive response to oestrogen deprivation; however, in this study, all appeared to converge on cell cycle regulation. Our data highlighting the relationship between the E2F signature and residual Ki67 along with the earlier proposal by Miller et al. [19] that on-treatment evaluation of this signature could indicate enhanced sensitivity to CDK4/6 inhibition suggests that it merits prospective evaluation in a clinical setting. This is a hypothesis that we will be testing in a major new national adjuvant trial, POETIC-A, in which patients with early ER+ breast cancer whose tumour continues to show high Ki67 expression after 2 weeks AI will be randomised to additional CDK4/6 inhibition or not.

## Supplementary information

**Additional file 1.** Supplementary information. Additional description of the materials and methods.

**Additional file 2.** **Table S1.** Published gene-signatures. **Table S2.** Study endpoints. **Table S3.** Study demographics. **Table S4.** List of patients and their status of HER2, Ki67 and CCCA.** Table S5.** Ki67 data summary. (A) Percentage change in Ki67 expression after 2-weeks AI-treatment. (B) Anti-proliferative response summary. (C) Summary of number of tumours that reached CCCA. Table S6. Genes whose baseline expression significantly correlated with Change.Ki67 (*p* < 0.005) in HER2- group. **Table S7.** Unpaired t-test of significance for the difference between two-group means: i) non-responders vs responders; ii) noCCCAs vs CCCAs in HER2- group. **Table S8.** Genes whose baseline expression significantly correlated with residual Ki67 (*p* < 0.005) in HER2- group. **Table S9.** Pathway analysis of genes in Table S8. Table S10. Genes whose baseline expression is significantly different (*p* < 0.001) between CCCA and noCCCA in HER2- group. **Table S11.** Genes significantly regulated by AI with *p* < 0.005 by univariate Two-sample T-test in HER2- group. **Table S12.** Top 20 AI-downregulated / AI-upregulated genes, and additional AI-regulated genes of interest identified from HER2- paired tumours. Genes were ranked by fold-change (FC). The Significantly different (*p*-value) derived from Mann-Whitney test comparing the relative change in expression of a given gene in the HER2- group versus the HER2+ group. ns: p >= 0.05. **Table S13.** Enriched canonical pathways of the significantly AI-regulated genes in Table S11. **Table S14.** Correlations of the change in ESR1 / gene-signatures and change in Ki67 / residual-Ki67 in i) HER2- group, ii) HER2+ group, iii) Significance of the difference between the 2 correlation-coefficients. **Table S15.** Genes significantly regulated by AI with *p* < 0.005 by univariate Two-sample T-test in HER2+ group. **Table S16.** Genes whose baseline expression significantly correlated with Change.Ki67 (*p* < 0.005) in HER2+ group. **Table S17.** Genes whose baseline expression significantly correlated with residual-Ki67 (*p* < 0.005) in HER2+ group.

**Additional file 3: Figure S1.** Consort diagram showing derivation of samples for microarray analysis and Ki67 measurement. In total, RNA was extracted from 861 RNAlater stored core-cuts and 605 RNA samples with RNA integrity number (RIN) >4 and RNA >500 ng were sent for profiling. Samples were excluded due to lack of adequate estradiol suppression, RIN < 4 when profiling, or due to gene expression data of poor quality. “Pairs” indicates a tumour with matched baseline and surgery expression data; B (baseline) indicates a tumour with baseline expression data or baseline Ki67 value; S (surgery) indicates a tumour with surgical expression data or on-treatment Ki67. *Expression QC (quality control): samples with fraction of detection rate <30% or detected by lumi.outlier function from lumi R package as outlier were excluded.

**Additional file 4: Figure S2.** Heatmap (Pearson, complete) of 123 genes whose baseline expression significantly correlated with Change.Ki67 (p<0.005) based on 155 HER2- of the 178 AI-treated samples. The gene expression across 155 samples was centred and scaled. Red denotes the gene expression in a sample is greater than the mean, blue denotes less than the mean. The tumours are ordered according to the degree of change in Ki67.

**Additional file 5: Figure S3.** Scatter plot of baseline gene expression and Ki67 values. (**a**) ESR1 baseline expression and change in Ki67 of HER2- tumours; (**b**) ESR1 baseline expression and residual Ki67 of HER2- tumours; (**c**) ESR1 baseline expression and change in Ki67 of HER2+ tumours; (**d**) ESR1 baseline expression and residual Ki67 of HER2+ tumours.

**Additional file 6: Figure S4.** Pathway analysis of the list of 123 genes whose baseline expression correlated with change in Ki67 in the HER2- tumours by Spearman correlation at a p-value of <0.005.

**Additional file 7: Figure S5.** Heatmap of Spearman Rank Correlation Matrix (r-value and p -value) of baseline gene signature scores and percentage of 2-week change in Ki67 and residual Ki67 expression. r-values bottom, *p*-values top. (**a**) HER2- tumours, *n*=155. (**b**) HER2+ tumours, *n*=23.

**Additional file 8: ** Matrices of correlation coefficient and *p* value. **Table S18.** Spearman Rank Correlation Matrix (r-value and p -value) of baseline gene signature scores and percentage of two-week change in Ki67 and residual Ki67 expression. r-values bottom, p-values top. (a) HER2- tumours, *n*=155 (the Signature name was sorted based on the rho-values of correlations between GS-score and residual.Ki67; (b) HER2+ tumours, *n*=23. **Table S19.** Spearman correlations (r-value and p -value) between on-treatment gene signature scores and i) percentage of two-week change in Ki67 protein expression and ii) residual KI67. r-values bottom, p-values top. (a) HER2- tumours, *n*=135; (b) HER2+ tumours, *n*=22. **Table S20.** Spearman correlations (r-value and p -value) between change in gene signature scores and i) percentage of two-week change in Ki67 protein expression and ii) residual KI67. r-values bottom, p-values top. (a) HER2- tumours, *n*=135; (b) HER2+ tumours, *n*=22.

**Additional file 9: Figure S6.** The 41 of the 902 significantly regulated genes after 2-week of treatment in HER2- tumours, that occurred in at least 3 of the significantly enriched 25 canonical pathways (FDR < 5%). Black-color and grey-color indicates presence and absence of a gene in a pathway. The numbers at the second row on the top of the black and grey image indicated number of pathways that a gene occurred. The numbers at the second column on the left-hand side of the black and grep image indicated number of genes occurred in a pathway.

**Additional file 10: Figure S7.** Heatmap of Spearman correlations (r-value and p -value) between on-treatment gene signature scores and i) percentage of 2-week change in Ki67 protein expression and ii) residual KI67. r-values bottom, p-values top. (**a**) HER2- tumours, *n*=135. (**b**) HER2+ tumours, *n*=22.

**Additional file 11: Figure S8.** Heatmap of Spearman correlations (r-value and p -value) between change in gene signature scores and i) percentage of 2-week change in Ki67 protein expression and ii) residual KI67. r-values bottom, p-values top. (**a**) HER2- tumours, *n*=135. (**b**) HER2+ tumours, *n*=22.

**Additional file 12: Figure S9.** Overrepresented pathways (FDR < 5%) identified by pathway analysis (IPA) of the 71 differentially expressed and annotated genes derived from HER2+ tumours. Negative z-score shown in blue-colour specifies inhibited pathway after AI-treatment. The yellow line indicates the threshold of adjusted p-value < 0.05.

## Data Availability

Global gene expression data supporting the finding from this manuscript was deposited at the NCBI gene expression omnibus (GEO) (http://ncbi.nlm.nih.gov/geo/) with accessions of GSE105777 and GSE126870.
