# Supplementary material for: Impact of aromatase inhibitor treatment on global gene expression and its association with antiproliferative response in ER+ breast cancer in postmenopausal patients
Source: Breast Cancer Res. 2019 Dec 31;22:2. doi: 10.1186/s13058-019-1223-z (PMC6938628; doi:10.1186/s13058-019-1223-z)
Supplement: Supplementary file 1 — Additional file 1. Supplementary information. Additional description of the materials and methods. [file 13058_2019_1223_MOESM1_ESM.docx]

**MATERIALS AND METHODS**

**Patients and samples**

The patients studied were a subpopulation of the POETIC (PeriOperative Endocrine Therapy for Individualised Care) study. The study randomised 4486 patients with primary ER+ breast cancer in 130 centres in UK to receive 2 weeks’ AI (letrozole or anastrozole) before surgery or no presurgical treatment (2:1). Core-cut biopsies were taken and fixed in formalin from all patients prior to randomised management and at surgery. At a minority of centres additional core-cuts were taken at the same time points and placed in RNAlater. These RNAlater fixed biopsies form the sample set for studying transcription in the current study.

**RNA extraction**

Total RNA was extracted using miRNeasy (Qiagen, Sussex, UK). RNA quality was checked using an Agilent Bioanalyser (Santa Clara, CA, USA). In total, RNA was extracted from 861 RNAlater stored core-cuts. 605 RNA samples with RNA integrity number (RIN) >4 and RNA >500 ng were sent for profiling. Patients were excluded when the estradiol levels at surgery were above 10pM (AI-treated patients) or baseline value was > 100pM. And 8 samples were excluded due to lack of adequate estradiol suppression (**Additional file 3: Fig. S1**).

**Ethics statement**

Ethical approval for POETIC (Trial Number CRUK/07/015) was provided by NRES Committee London –South East. All patients consented to molecular analysis of their samples for research purposes.

**Gene expression analysis and data pre-processing**

RNA amplification, labelling and hybridization on HumanHT-12_V4 expression BeadChips (Illumina, San Diego, CA, USA) were performed, according to the manufacturer's instructions. The raw data was extracted using GenomeStudio Software and was processed in R using lumi package (<http://www.bioconductor.org>).

In brief, data was (i) filtered to remove any non-expressed probes (detection p > 0.01) across samples in corresponding dataset, (ii) transformed using the variance-stabilising transformation, (iii) normalised using the robust spline normalisation method, and (iv) batch-corrected using the function (ComBat) in the R package (sva). Samples were excluded if their fraction of detected genes was < 30% (**Additional file 3: Fig. S1**) or identified as outliers by a sample outlier detection function in the lumi package (**Additional file 3: Fig. S1**). Probes were further filtered out if they were not detected in at least 25% of the paired samples or in at least 75% of the baseline samples. When multiple probes mapped to the same gene, the most variable probe measured by interquartile range across samples was selected to represent the gene. Gene expression data from this study is deposited at GEO with accessions of GSE105777 and GSE126870.

**Elimination of gene expression changes in Control group**

To correct for potential artifactual changes in gene expression that resulted from study procedures [1], the 2-week changes in expression resulting from AI treatment was estimated for each gene by comparing the expression changes (log_2_^(Surgery/Baseline)^) in the AI-treated tumours and the expression changes (log_2_^(Surgery/Baseline)^) of the un-treated tumours. The relative (corrected) gene expression level in a given sample was calculated by subtracting the mean expression for the gene in the control samples from the expression of the given gene in the AI-treated tumour. All data shown that relate to either on-treatment expression/signature-score or changes in expression/signature-score were corrected in this manner.

**Biomarker analyses**

Ki67 (%) staining on formalin-fixed samples was carried out using anti-MIB-1 (M7240, DAKO UK), and analysed centrally, as previously described [2]. HER2 status was measured locally using immunohistochemistry and/or in situ Hybridization [3].

**Published Gene Signatures**

Previously, we reported the association of several key biological processes represented by a series of gene signatures that associate with response to AI-therapy [4]. The same panel (**Additional file 2: Table S1**) was assessed in this study together with the following published signatures with their name in italics to further interrogate putative markers of response or resistance: signatures representing the PI3K pathway (*PI3K-GS*) [5], two loss of the retinoblastoma gene signatures (*RBloss-GS, DiLeoRBloss-GS*) [6], target genes of ER (*ERTarget-GS*) and Wnt (*WntTarget-GS*) pathway [7]; *E2Factivation-GS:* an E2F activity signature excluding cell cycle genes that were identified by their correlation with mean expression of 24 genes; the signature had previously been found to be significantly associated with residual Ki67 level in ER+ tumours after estrogen deprivation with AI [8]; *TP53-GS:* a 39-gene p53 signature derived from comparison of p53-wild-type ER+ tumours versus p53-mutant ER+ tumours (1) [9]; *Bcell-GS, Tcell-GS and MacTh1-GS:* signatures related to specific immune cell subsets, a 23-gene signature specific to B-cells, a 83-gene signature specific to T-cells and a 105-gene signature specific to macrophages [10]; *Inflammatory-GS*: a 45-gene signature enriched in dendritic cells and containing a transcriptional fingerprint of infiltrating immune cells [11]; *E2F4*-*GS*: an E2F4 target activation signature including 24 genes whose expression were significantly upregulated in letrozole-resistant tumors, but suppressed by CDK4/6 inhibition in estrogen-deprived ER+ breast cancer cells and in patients' ER+ tumors [12]; *GDNF-GS*, a proliferation-independent GDNF response signature reported to be prognostic of poor patient outcome and poor response to AI treatment with the development of resistance in ER+ breast cancers [13].

**Immune or Stromal Score Estimation**

To allow comparison of the extent of immune or stromal admixture between samples, we used ESTIMATE [14]. This consists of two gene signatures incorporating information from 141-immune associated genes or 141-stromal genes. In brief, based on the normalized log_2_-expression, we used single sample gene set enrichment analysis (ssGSEA) from R-package (ESTIMATE) to determine the proportion of immune or stroma cell content within the samples.

**Statistical analysis**

Unpaired T-tests were used to compare the mean changes in gene expression (log_2_^(Surgery/Baseline)^) of tumours in the Treated versus the Control group using BRB-Array Tools (<https://brb.nci.nih.gov/BRB-ArrayTools/>). The Ingenuity Pathways Analysis (IPA) was conducted on the lists of genes that associated with change in Ki67, or residual Ki67, or differentially expressed to identify over-represented pathways. Pathways were considered to be statistically enriched when false discovery rate (FDR) < 5%; the association between two groups was considered to be statistically significant when p-value <0.005; the difference between two groups considered to be statistically significant when p-value <0.001. Otherwise, indicated in the figure legends or in the main text. Reported p-values are two-sided.

To visualise the degree of variability between the tumours in their transcriptional response to estrogen deprivation we performed hierarchical clustering with Pearson correlation and ward.D2 method, using the values representing the relative change in expression/signature-score of each of the regulated genes upon AI-treatment; and tumours were sorted by the residual (2-week) Ki67 value.

The signature-scores were estimated as the weighted average values as previously described [4].

Four endpoints were used in this study: (i) change in Ki67 between baseline and 2-weeks as a continuous variable and (ii) responder or non-responder, defined as a reduction of >60% or <60%, respectively, in Ki67 [15]; (iii) residual (2-week) Ki67 as a continuous variable; (iv) presence or absence of complete cell cycle arrest (CCCA or noCCCA), ie. 2-week Ki67 <2.7% or >2.7%, respectively [16]. Each of the end-points provides different information: (i) and (ii) reflect the antiproliferative response to AI treatment which relates to benefit from the treatment, and end-points (iii) and (iv) relate to the residual risk after AI-therapy. Patients with a baseline Ki67 value <5% were excluded from (i) and (ii) because low pretreatment values can lead to highly aberrant estimates of proportional change.

Percentage change in Ki67 at 2 weeks was defined as: ((surgery.Ki67- baseline.Ki67)/baseline.Ki67)*100. The natural logarithm of the Ki67 value was calculated as LN(Ki67+0.1) and natural logarithm of the change in Ki67 was calculated as: LN^((surgery.Ki67+0.1)/(baseline.Ki67+0.1))^ which is equivalent to LN^(surgery.Ki67+0.1)^ - LN^(baseline.Ki67+0.1)^. The addition of the 0.1 values was to avoid LN of zero.

Geometric means of Ki67 of baseline or surgery tumours were calculated as: EXP^(AVERAGE(natural logarithm of Ki67 values))^.

Other statistics were performed using GraphPad Prism 6. D'Agostino test was used to assess the assumption of normality in each group. F-test was used to assess the equality of variances of two groups. Comparisons between 2 groups used the unpaired Student's t-test; Welch’s correction was applied if the 2 groups did not have equal variances. Nonparametric Mann-Whitney test was used if the data did not pass normality test with in a group. Wilcoxon matched-pairs signed rank test was used to test the change in expression/signature-score of pre-selected genes/signatures between baseline and surgery for the tumours reached CCCA or noCCCA.

Cluster analysis was performed using R version 3.4.1 (<https://www.bioconductor.org/>). Spearman's rank correlation was used to assess associations between the gene expression/pre-selected signature-score and residual Ki67 and the change in Ki67 after AI-therapy. All p-values reported were two tailed, with p< 0.05 considered as significant or as indicated in the text and figure legends. R-package cocor [17] was used to test of significance for the difference between two correlation coefficients of 2 independent groups.

Each tumour was classified into one of intrinsic subtypes, namely, Luminal A, Luminal B, HER2-Enriched, Basal-like or Normal-like according to PAM50 classifier [18].

REFERENCES:

1. Qiong Gao ELo-K, Maggie Chon U Cheang, James Morden, Ricardo Ribas, Kally Sidhu, David Evans, Vera Martins, Andrew Dodson,, Anthony Skene CH, Elizabeth Mallon, Abigail Evans, Judith M. Bliss, John Robertson, Ian Smith, Lesley-Ann Martin, Mitch Dowsett; on behalf of the POETIC Trial Management Group and Trialists. Major Impact of Sampling Methodology on Gene Expression in Estrogen Receptor–Positive Breast Cancer. JNCI Cancer Spectrum 2018;Volume 2(Issue 2):pky005 doi https://doi.org/10.1093/jncics/pky005

2. Dowsett M, Smith IE, Ebbs SR, Dixon JM, Skene A, A'Hern R, et al. Prognostic value of Ki67 expression after short-term presurgical endocrine therapy for primary breast cancer. Journal of the National Cancer Institute 2007;99(2):167-70 doi 10.1093/jnci/djk020.

3. Wolff AC, Hammond ME, Hicks DG, Dowsett M, McShane LM, Allison KH, et al. Recommendations for human epidermal growth factor receptor 2 testing in breast cancer: American Society of Clinical Oncology/College of American Pathologists clinical practice guideline update. Journal of clinical oncology : official journal of the American Society of Clinical Oncology 2013;31(31):3997-4013 doi 10.1200/JCO.2013.50.9984.

4. Gao Q, Patani N, Dunbier AK, Ghazoui Z, Zvelebil M, Martin LA, et al. Effect of aromatase inhibition on functional gene modules in estrogen receptor-positive breast cancer and their relationship with antiproliferative response. Clin Cancer Res 2014;20(9):2485-94 doi 10.1158/1078-0432.CCR-13-2602.

5. Creighton CJ, Fu X, Hennessy BT, Casa AJ, Zhang Y, Gonzalez-Angulo AM, et al. Proteomic and transcriptomic profiling reveals a link between the PI3K pathway and lower estrogen-receptor (ER) levels and activity in ER+ breast cancer. Breast Cancer Res 2010;12(3):R40 doi 10.1186/bcr2594.

6. Malorni L, Piazza S, Ciani Y, Guarducci C, Bonechi M, Biagioni C, et al. A gene expression signature of retinoblastoma loss-of-function is a predictive biomarker of resistance to palbociclib in breast cancer cell lines and is prognostic in patients with ER positive early breast cancer. Oncotarget 2016;7(42):68012-22 doi 10.18632/oncotarget.12010.

7. Verhaegh W, van Ooijen H, Inda MA, Hatzis P, Versteeg R, Smid M, et al. Selection of personalized patient therapy through the use of knowledge-based computational models that identify tumor-driving signal transduction pathways. Cancer research 2014;74(11):2936-45 doi 10.1158/0008-5472.CAN-13-2515.

8. Miller TW, Balko JM, Fox EM, Ghazoui Z, Dunbier A, Anderson H, et al. ERalpha-dependent E2F transcription can mediate resistance to estrogen deprivation in human breast cancer. Cancer discovery 2011;1(4):338-51 doi 10.1158/2159-8290.CD-11-0101.

9. Coutant C, Rouzier R, Qi Y, Lehmann-Che J, Bianchini G, Iwamoto T, et al. Distinct p53 gene signatures are needed to predict prognosis and response to chemotherapy in ER-positive and ER-negative breast cancers. Clin Cancer Res 2011;17(8):2591-601 doi 10.1158/1078-0432.CCR-10-1045.

10. Iglesia MD, Vincent BG, Parker JS, Hoadley KA, Carey LA, Perou CM, et al. Prognostic B-cell signatures using mRNA-seq in patients with subtype-specific breast and ovarian cancer. Clin Cancer Res 2014;20(14):3818-29 doi 10.1158/1078-0432.CCR-13-3368.

11. Dunbier AK, Ghazoui Z, Anderson H, Salter J, Nerurkar A, Osin P, et al. Molecular profiling of aromatase inhibitor-treated post-menopausal breast tumors identifies immune-related correlates of resistance. Clin Cancer Res 2013 doi 10.1158/1078-0432.CCR-12-1000.

12. Guerrero-Zotano AL, Stricker TP, Formisano L, Hutchinson KE, Stover DG, Lee KM, et al. ER(+) Breast Cancers Resistant to Prolonged Neoadjuvant Letrozole Exhibit an E2F4 Transcriptional Program Sensitive to CDK4/6 Inhibitors. Clin Cancer Res 2018;24(11):2517-29 doi 10.1158/1078-0432.CCR-17-2904.

13. Morandi A, Martin LA, Gao Q, Pancholi S, Mackay A, Robertson D, et al. GDNF-RET signaling in ER-positive breast cancers is a key determinant of response and resistance to aromatase inhibitors. Cancer research 2013;73(12):3783-95 doi 10.1158/0008-5472.CAN-12-4265.

14. Yoshihara K, Shahmoradgoli M, Martinez E, Vegesna R, Kim H, Torres-Garcia W, et al. Inferring tumour purity and stromal and immune cell admixture from expression data. Nat Commun 2013;4:2612 doi 10.1038/ncomms3612.

15. Gellert P, Segal CV, Gao Q, Lopez-Knowles E, Martin LA, Dodson A, et al. Impact of mutational profiles on response of primary oestrogen receptor-positive breast cancers to oestrogen deprivation. Nat Commun 2016;7:13294 doi 10.1038/ncomms13294.

16. Ma CX, Gao F, Luo J, Northfelt DW, Goetz M, Forero A, et al. NeoPalAna: Neoadjuvant Palbociclib, a Cyclin-Dependent Kinase 4/6 Inhibitor, and Anastrozole for Clinical Stage 2 or 3 Estrogen Receptor-Positive Breast Cancer. Clin Cancer Res 2017 doi 10.1158/1078-0432.CCR-16-3206.

17. Diedenhofen B, Musch J. cocor: a comprehensive solution for the statistical comparison of correlations. PLoS One 2015;10(3):e0121945 doi 10.1371/journal.pone.0121945.

18. Parker JS, Mullins M, Cheang MCU, et al. Supervised risk predictor of breast cancer based on intrinsic subtypes, J Clin Oncol , 2009, vol. 27 (pg. 1160-1167).
