# Supplementary material for: Impact of aromatase inhibitor treatment on global gene expression and its association with antiproliferative response in ER+ breast cancer in postmenopausal patients
Source: Breast Cancer Res. 2019 Dec 31;22:2. doi: 10.1186/s13058-019-1223-z (PMC6938628; doi:10.1186/s13058-019-1223-z)
Supplement: Supplementary file 3 — Additional file 3: Figure S1. Consort diagram showing derivation of samples for microarray analysis and Ki67 measurement. In total, RNA was extracted from 861 RNAlater stored core-cuts and 605 RNA samples with RNA integrity number (RIN) >4 and RNA >500 ng were sent for profiling. Samples were excluded due to lack of adequate estradiol suppression, RIN < 4 when profiling, or due to gene expression data of poor quality. “Pairs” indicates a tumour with matched baseline and surgery expression data; B (baseline) indicates a tumour with baseline expression data or baseline Ki67 value; S (surgery) indicates a tumour with surgical expression data or on-treatment Ki67. *Expression QC (quality control): samples with fraction of detection rate <30% or detected by lumi.outlier function from lumi R package as outlier were excluded. [file 13058_2019_1223_MOESM3_ESM.pdf]

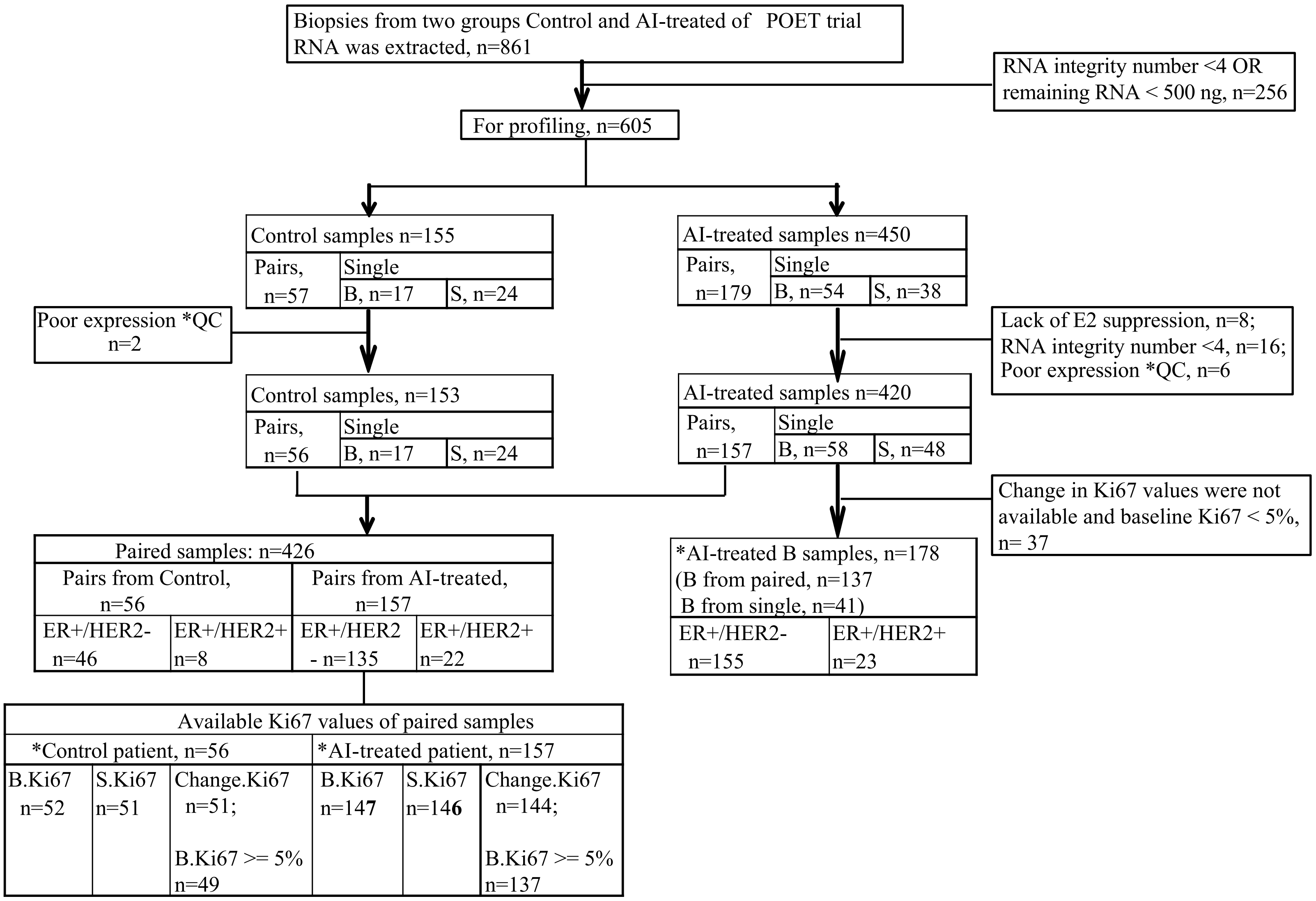

* Number of patients' data used in the study from

- no presurgical treatment group, n = 56
- 2 week's presurgical AI group : n = 198 (157 + 41)

Additional file 3: Figure S1
