# Supplementary figures and images for: Impact of aromatase inhibitor treatment on global gene expression and its association with antiproliferative response in ER+ breast cancer in postmenopausal patients
Source: Breast Cancer Res. 2019 Dec 31;22:2. doi: 10.1186/s13058-019-1223-z (PMC6938628; doi:10.1186/s13058-019-1223-z)

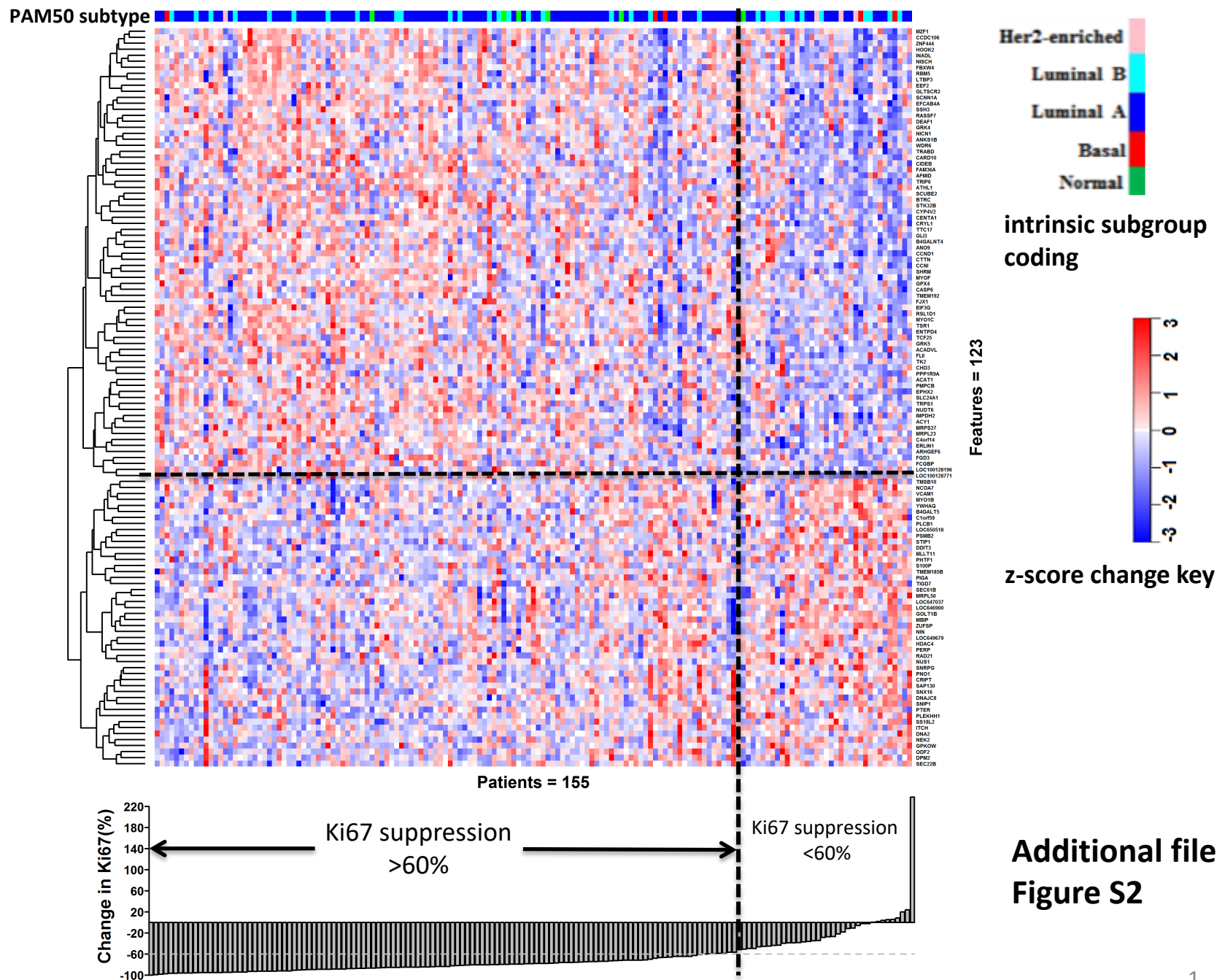

Supplement: Supplementary file 4 — Additional file 4: Figure S2. Heatmap (Pearson, complete) of 123 genes whose baseline expression significantly correlated with Change.Ki67 (p<0.005) based on 155 HER2- of the 178 AI-treated samples. The gene expression across 155 samples was centred and scaled. Red denotes the gene expression in a sample is greater than the mean, blue denotes less than the mean. The tumours are ordered according to the degree of change in Ki67. [file 13058_2019_1223_MOESM4_ESM.pdf]

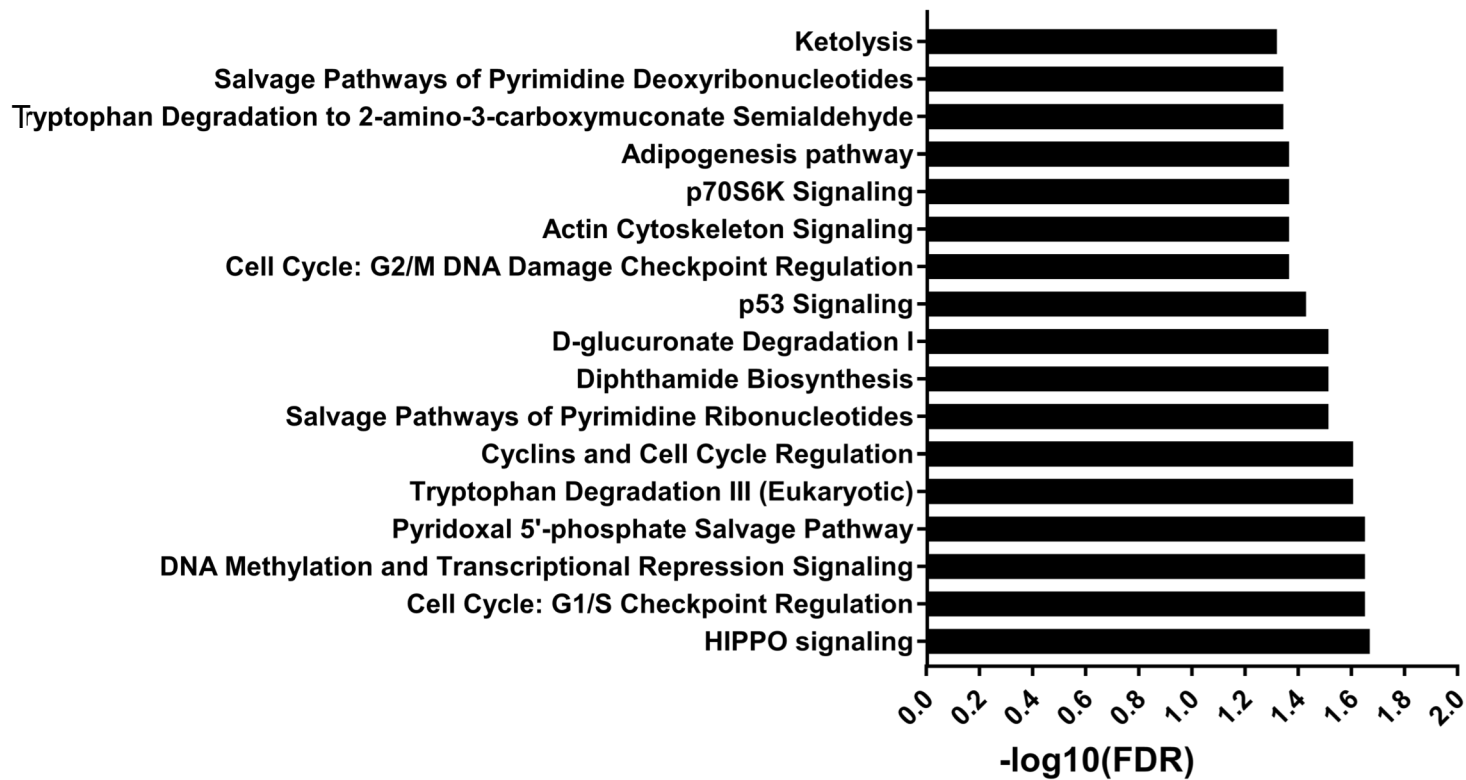

**Additional file 6:  
Figure. S4**

Supplement: Supplementary file 6 — Additional file 6: Figure S4. Pathway analysis of the list of 123 genes whose baseline expression correlated with change in Ki67 in the HER2- tumours by Spearman correlation at a p-value of <0.005. [file 13058_2019_1223_MOESM6_ESM.pdf]

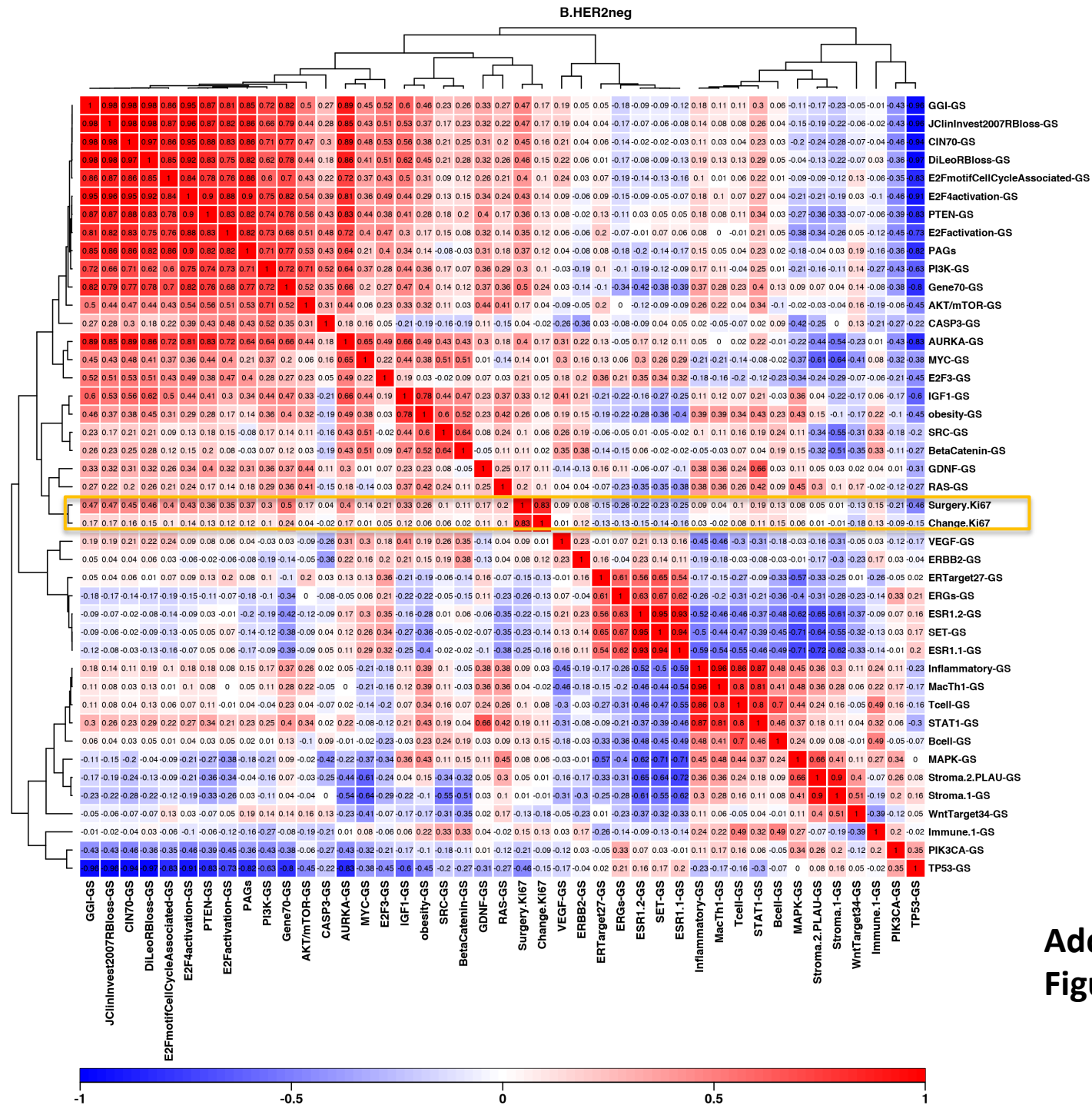

**Additional file 7:  
Figure S5a**

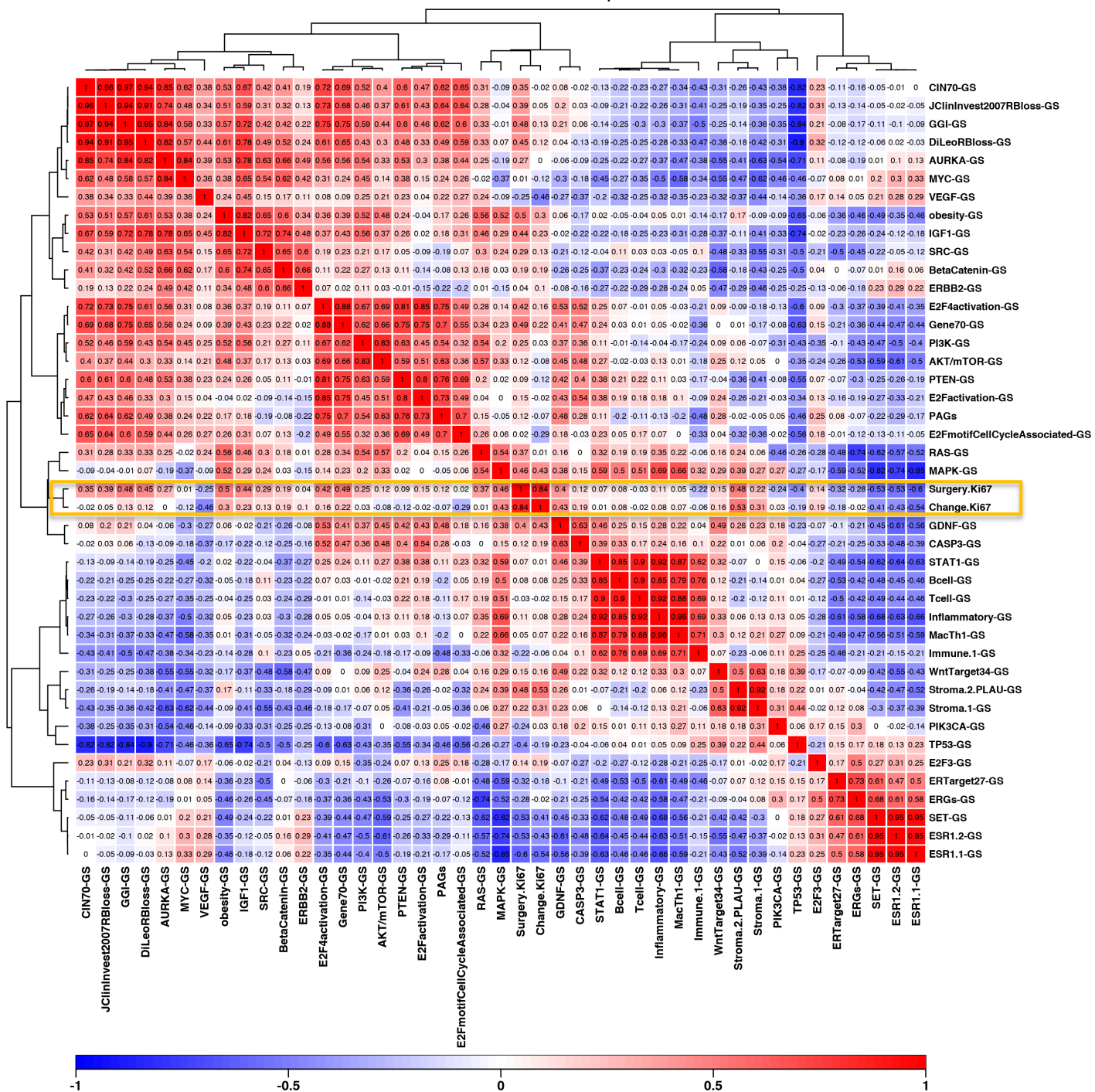

Supplement: Supplementary file 7 — Additional file 7: Figure S5. Heatmap of Spearman Rank Correlation Matrix (r-value and p -value) of baseline gene signature scores and percentage of 2-week change in Ki67 and residual Ki67 expression. r-values bottom, p-values top. (a) HER2- tumours, n=155. (b) HER2+ tumours, n=23. [file 13058_2019_1223_MOESM7_ESM.pdf]

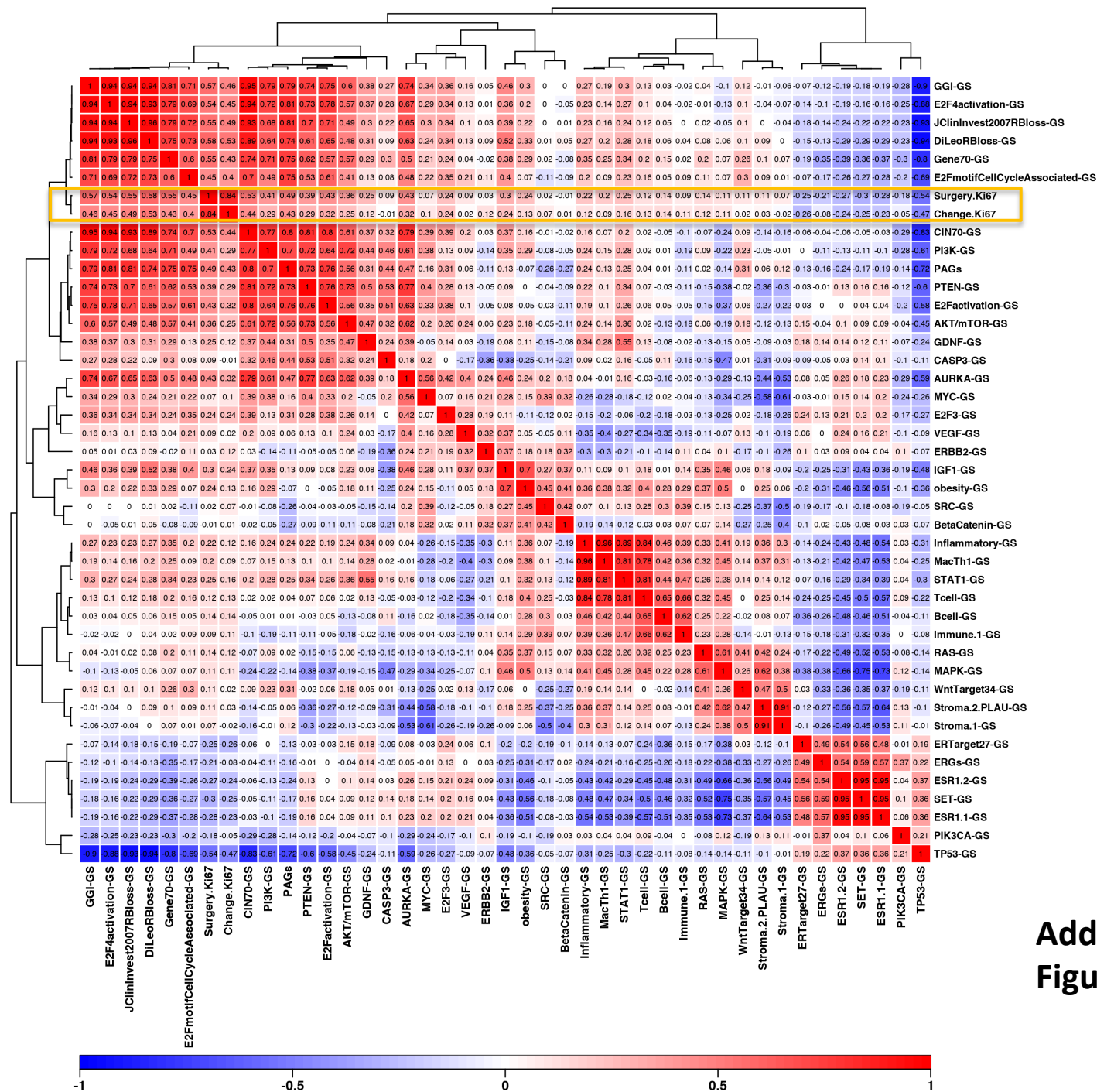

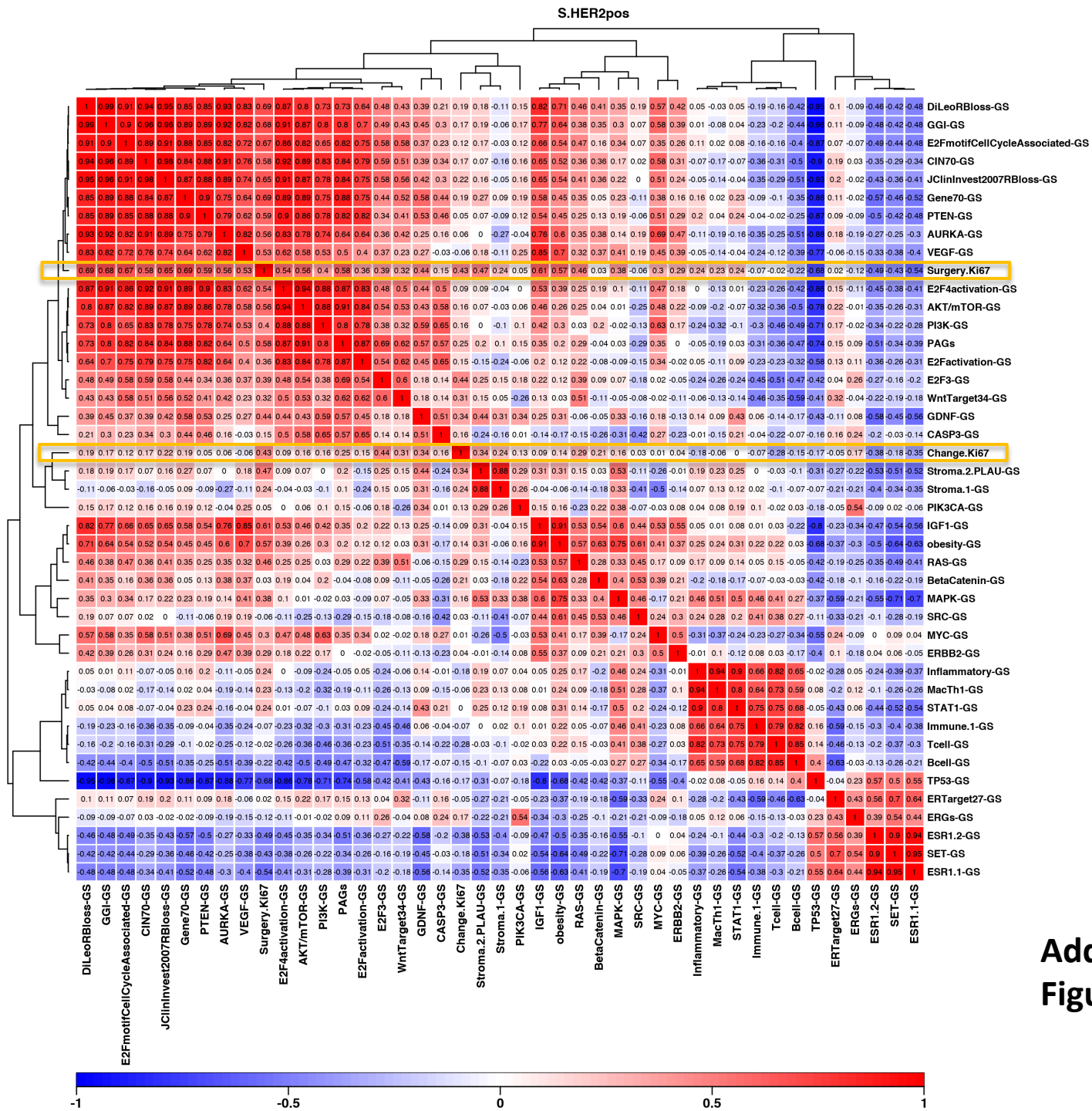

Additional file 10:  
Figure S7b

Supplement: Supplementary file 10 — Additional file 10: Figure S7. Heatmap of Spearman correlations (r-value and p -value) between on-treatment gene signature scores and i) percentage of 2-week change in Ki67 protein expression and ii) residual KI67. r-values bottom, p-values top. (a) HER2- tumours, n=135. (b) HER2+ tumours, n=22. [file 13058_2019_1223_MOESM10_ESM.pdf]

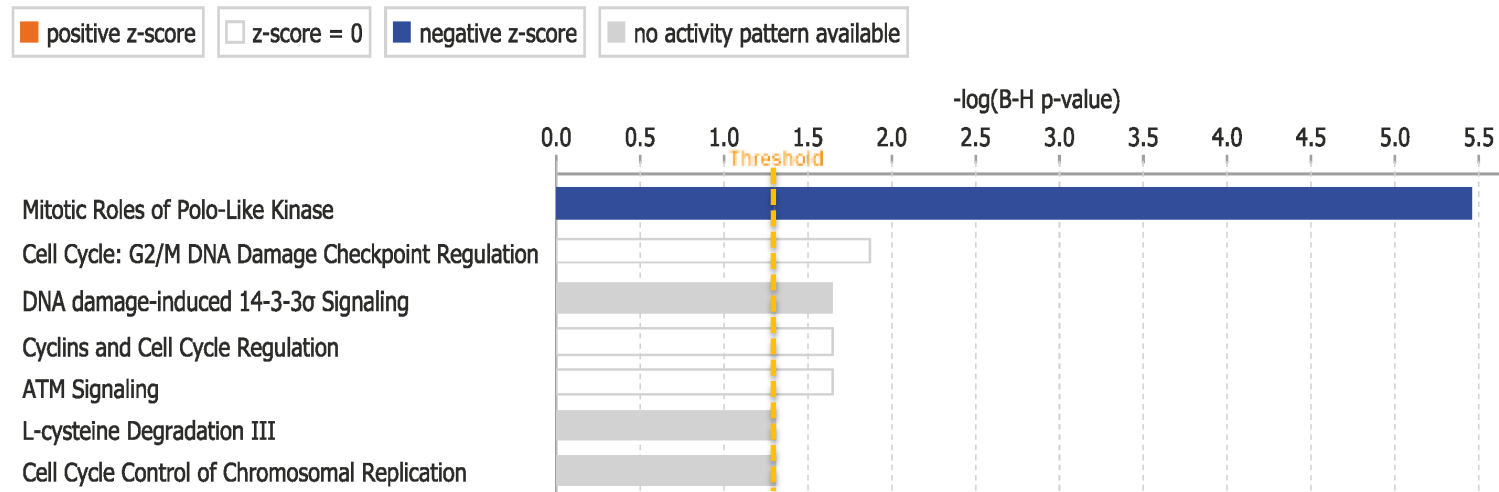

**Additional file 12:  
Figure S9**

Supplement: Supplementary file 12 — Additional file 12: Figure S9. Overrepresented pathways (FDR < 5%) identified by pathway analysis (IPA) of the 71 differentially expressed and annotated genes derived from HER2+ tumours. Negative z-score shown in blue-colour specifies inhibited pathway after AI-treatment. The yellow line indicates the threshold of adjusted p-value < 0.05. [file 13058_2019_1223_MOESM12_ESM.pdf]
