# Supplementary material for: Impact of aromatase inhibitor treatment on global gene expression and its association with antiproliferative response in ER+ breast cancer in postmenopausal patients
Source: Breast Cancer Res. 2019 Dec 31;22:2. doi: 10.1186/s13058-019-1223-z (PMC6938628; doi:10.1186/s13058-019-1223-z)
Supplement: Supplementary file 5 — Additional file 5: Figure S3. Scatter plot of baseline gene expression and Ki67 values. (a) ESR1 baseline expression and change in Ki67 of HER2- tumours; (b) ESR1 baseline expression and residual Ki67 of HER2- tumours; (c) ESR1 baseline expression and change in Ki67 of HER2+ tumours; (d) ESR1 baseline expression and residual Ki67 of HER2+ tumours. [file 13058_2019_1223_MOESM5_ESM.pdf]

a

POETIC HER2- (n=155) baselineESR1 change in Ki67

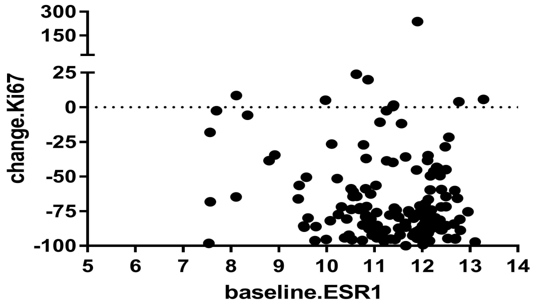

	baseline.ESR1 vs. change.Ki67
Spearman r	
r	-0.1099
95% confidence interval	-0.2673 to 0.0533
P value	
P (two-tailed)	0.1735
P value summary	ns
Exact or approximate P value?	Approximate
Significant? (alpha = 0.05)	No
Number of XY Pairs	155

c

POETIC HER2+ (n=23) baselineESR1 change in Ki67

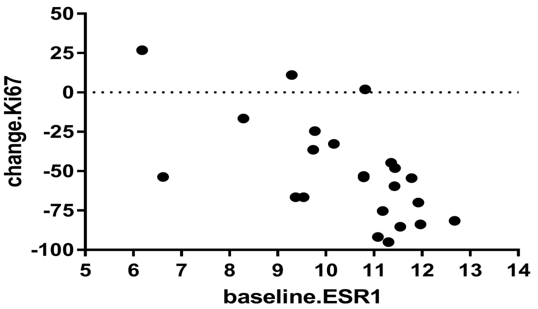

	baseline.ESR1 vs. change.Ki67
Spearman r	
r	-0.6077
95% confidence interval	-0.8199 to -0.2487
P value	
P (two-tailed)	0.0021
P value summary	**
Exact or approximate P value?	Approximate
Significant? (alpha = 0.05)	Yes
Number of XY Pairs	23

b

POETIC HER2- (n=155) baselineESR1 residual Ki67

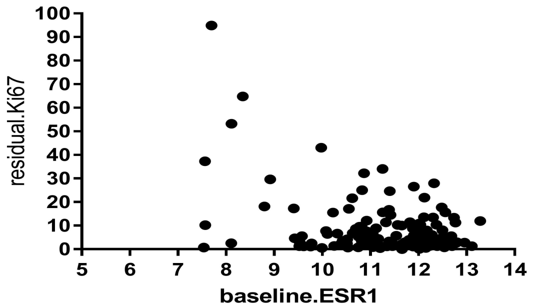

	baseline.ESR1 vs. residual.Ki67
Spearman r	
r	-0.1558
95% confidence interval	-0.3102 to 0.00657
P value	
P (two-tailed)	0.0529
P value summary	ns
Exact or approximate P value?	Approximate
Significant? (alpha = 0.05)	No
Number of XY Pairs	155

d

POETIC HER2+ (n=23) baselineESR1 residual Ki67

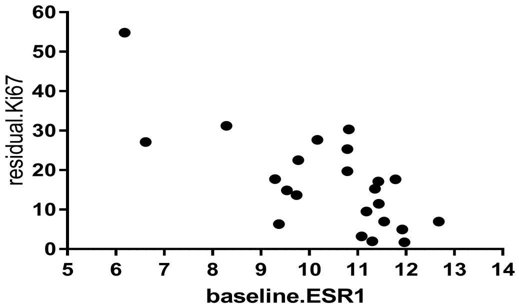

	baseline.ESR1 vs. residual.Ki67
Spearman r	
r	-0.6156
95% confidence interval	-0.824 to -0.2605
P value	
P (two-tailed)	0.0018
P value summary	**
Exact or approximate P value?	Approximate
Significant? (alpha = 0.05)	Yes
Number of XY Pairs	23

Additional file 5: Figure S3
