# Supplementary material for: Impact of aromatase inhibitor treatment on global gene expression and its association with antiproliferative response in ER+ breast cancer in postmenopausal patients
Source: Breast Cancer Res. 2019 Dec 31;22:2. doi: 10.1186/s13058-019-1223-z (PMC6938628; doi:10.1186/s13058-019-1223-z)
Supplement: Supplementary file 9 — Additional file 9: Figure S6. The 41 of the 902 significantly regulated genes after 2-week of treatment in HER2- tumours, that occurred in at least 3 of the significantly enriched 25 canonical pathways (FDR < 5%). Black-color and grey-color indicates presence and absence of a gene in a pathway. The numbers at the second row on the top of the black and grey image indicated number of pathways that a gene occurred. The numbers at the second column on the left-hand side of the black and grep image indicated number of genes occurred in a pathway. [file 13058_2019_1223_MOESM9_ESM.pdf]

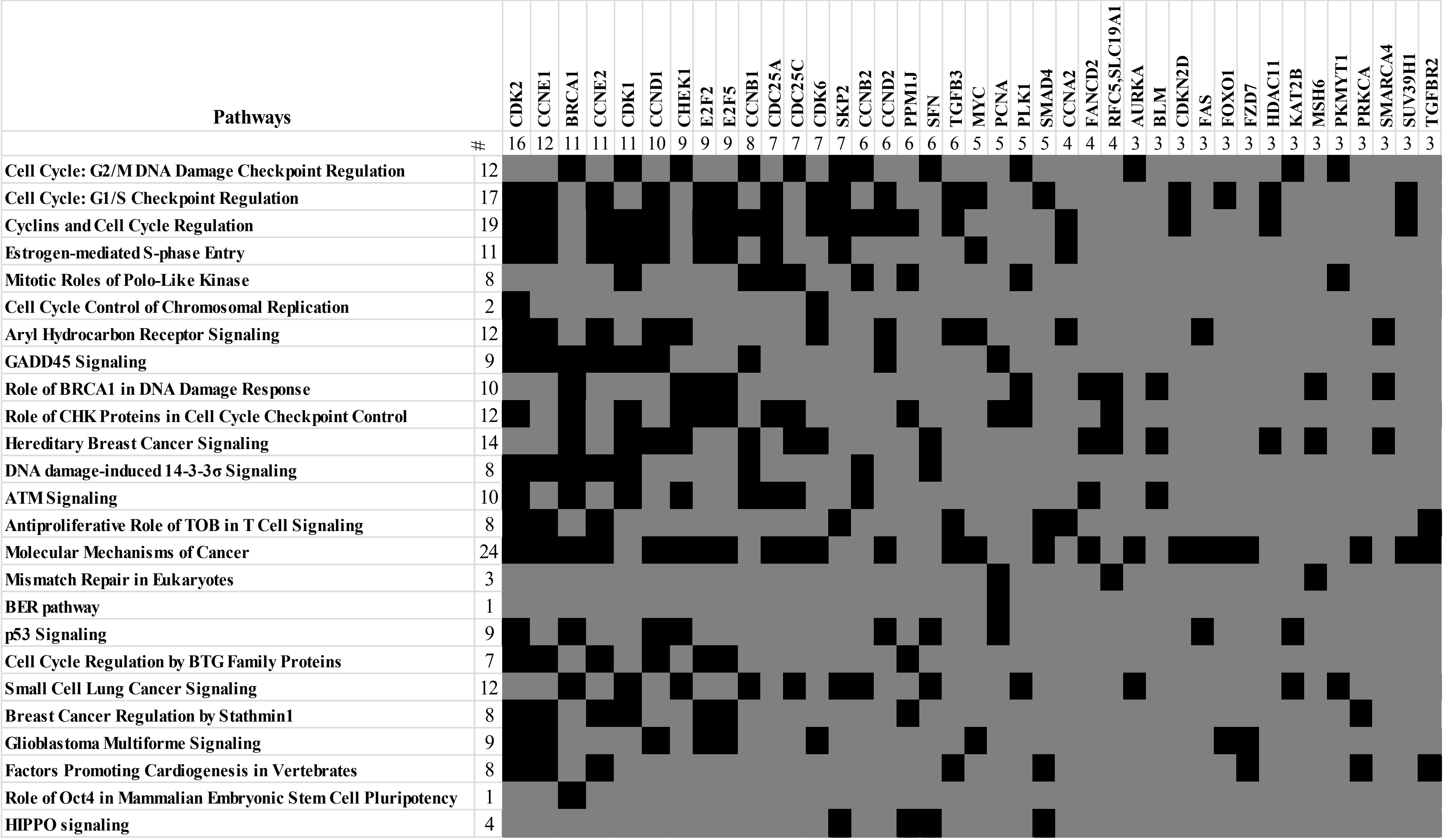

Additional file 9: Figure S6
