# Supplementary material for: Impact of aromatase inhibitor treatment on global gene expression and its association with antiproliferative response in ER+ breast cancer in postmenopausal patients
Source: Breast Cancer Res. 2019 Dec 31;22:2. doi: 10.1186/s13058-019-1223-z (PMC6938628; doi:10.1186/s13058-019-1223-z)
Supplement: Supplementary file 11 — Additional file 11: Figure S8. Heatmap of Spearman correlations (r-value and p -value) between change in gene signature scores and i) percentage of 2-week change in Ki67 protein expression and ii) residual KI67. r-values bottom, p-values top. (a) HER2- tumours, n=135. (b) HER2+ tumours, n=22. [file 13058_2019_1223_MOESM11_ESM.pdf]

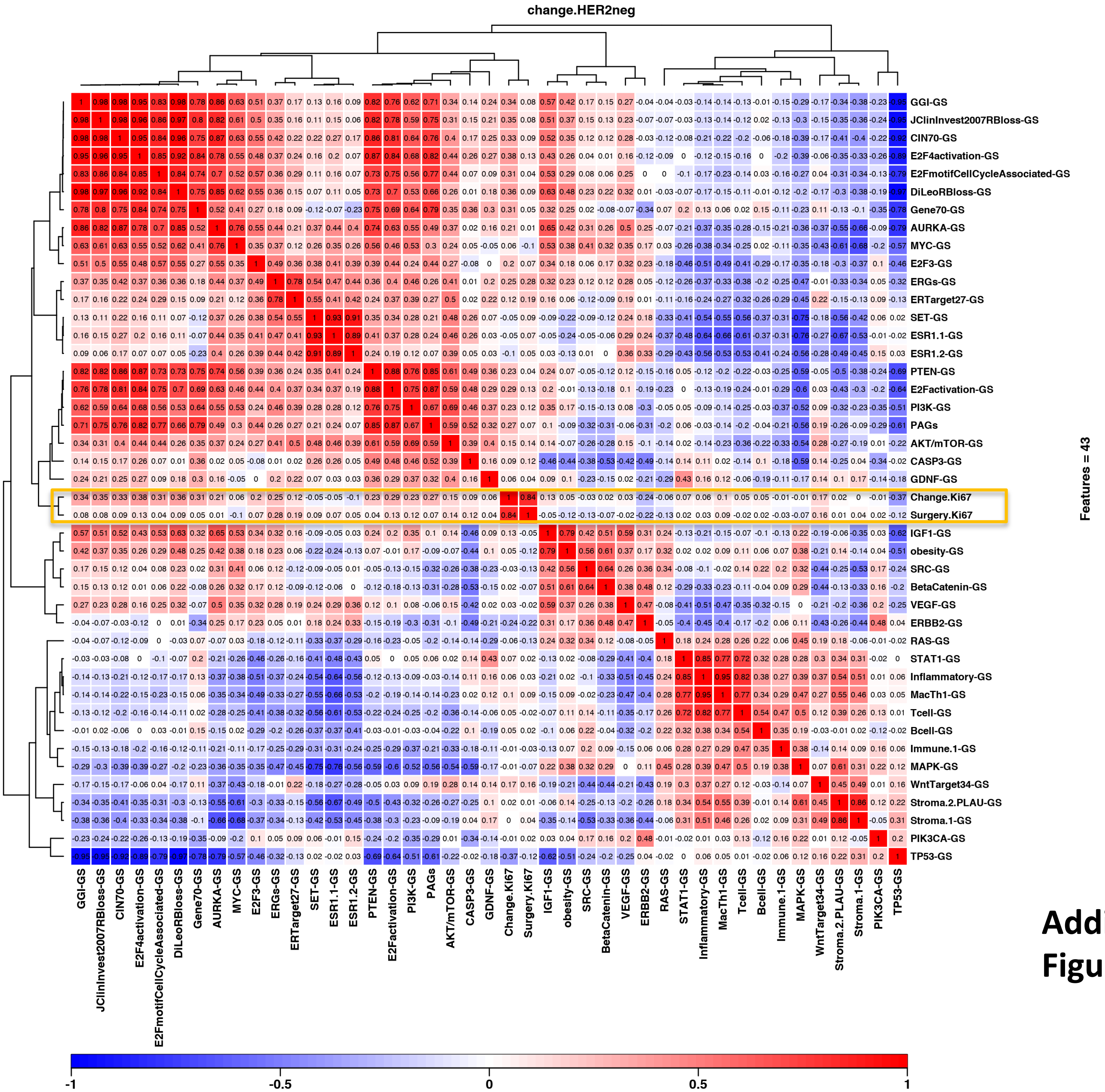

Additional file 11: Figure S8a

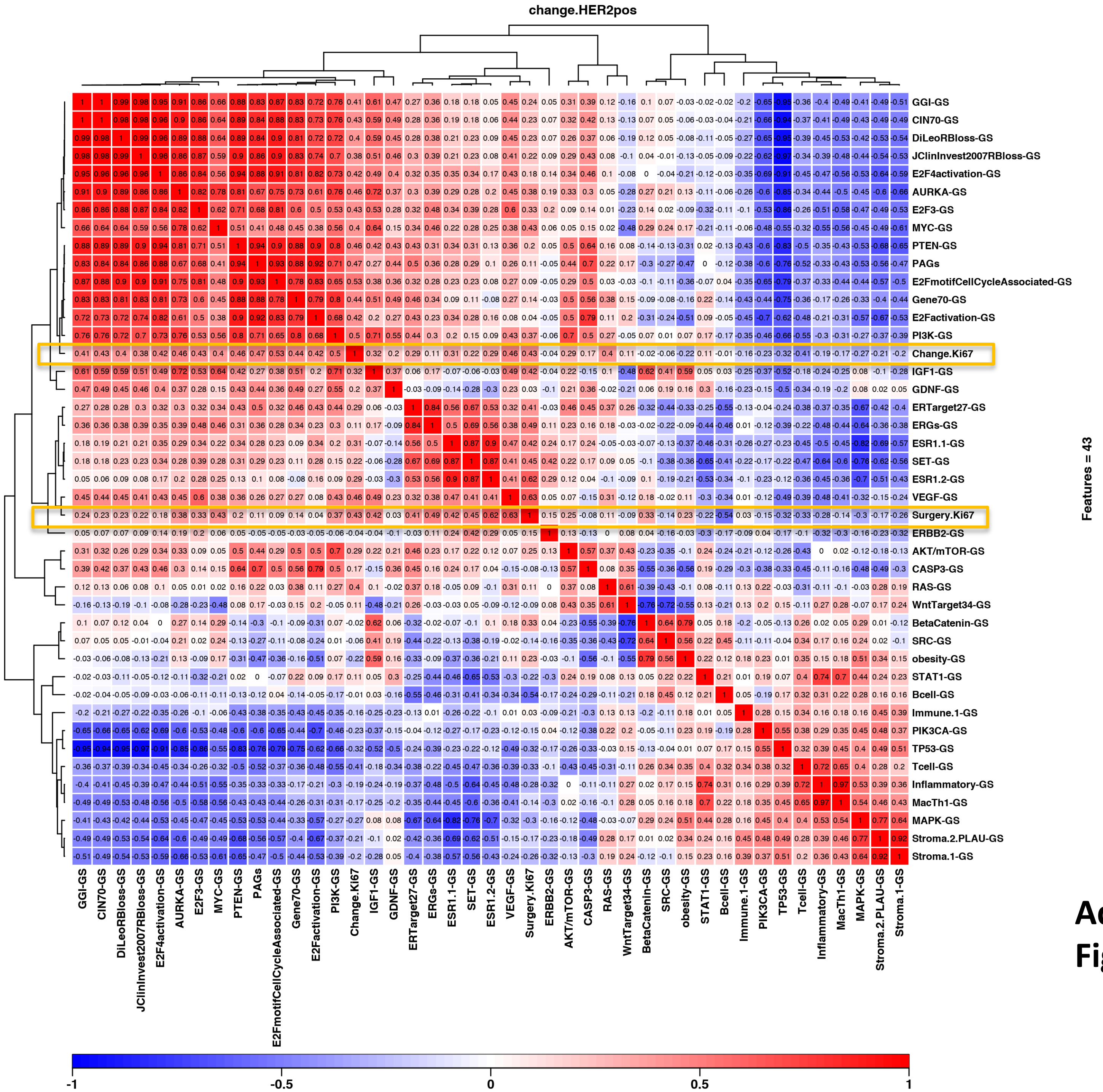

Additional file 11: Figure S8b
